# Genomic patterns of diversity and divergence of two introduced salmonid species in Patagonia, South America

**DOI:** 10.1111/eva.12464

**Published:** 2017-03-06

**Authors:** Shawn R. Narum, Pablo Gallardo, Cristian Correa, Amanda Matala, Daniel Hasselman, Ben J. G. Sutherland, Louis Bernatchez

**Affiliations:** ^1^ Hagerman Fish Culture Experiment StationColumbia River Inter‐Tribal Fish CommissionHagermanIDUSA; ^2^ Centro de Cultivos Marinos Bahía LaredoUniversity of MagallanesPunta ArenasChile; ^3^ Facultad de Ciencias Forestales y Recursos NaturalesInstituto de Conservación Biodiversidad y TerritorioUniversidad Austral de ChileValdiviaChile; ^4^ Facultad de CienciasInstituto de Ciencias Marinas y LimnológicasUniversidad Austral de ChileValdiviaChile; ^5^ Institut de Biologie Intégrative et des Systèmes (IBIS)Université LavalQuébecQCCanada

**Keywords:** adaptation, genomics, invasive species, RAD‐seq, salmonids

## Abstract

Invasive species have become widespread in aquatic environments throughout the world, yet there are few studies that have examined genomic variation of multiple introduced species in newly colonized environments. In this study, we contrast genomic variation in two salmonid species (anadromous Chinook Salmon, *Oncorhynchus tshawytscha*, 11,579 SNPs and resident Brook Charr *Salvelinus fontinalis*, 13,522 SNPs) with differing invasion success after introduction to new environments in South America relative to populations from their native range in North America. Estimates of genetic diversity were not significantly different between introduced and source populations for either species, indicative of propagule pressure that has been shown to maintain diversity in founding populations relative to their native range. Introduced populations also demonstrated higher connectivity and gene flow than those in their native range. Evidence for candidate loci under divergent selection was observed, but was limited to specific introduced populations and was not widely evident. Patterns of genomic variation were consistent with general dispersal potential of each species and therefore also the notion that life history variation may contribute to both invasion success and subsequent genetic structure of these two salmonids in Patagonia.

## 1 Introduction

In the last century, non‐native aquatic plant and animal species have become widely distributed throughout the world, primarily through human‐mediated activities (Cohen & Carlton, 1998; Roman & Darling, 2007). While many species have been dispersed accidentally (e.g., fish that escape from aquaculture or aquatic invertebrates transported in ship ballast tanks), some organisms are intentionally introduced for various objectives such as pest control, recreational fishing, or harvest opportunities. Introduced species that become established in non‐native locations can alter ecosystem dynamics in a manner that negatively impacts native species (Lodge, 1993), and many spread invasively to new locations. This may lead to local extirpation of native species and complications for water resource users, resulting in urgency to balance human activities with requirements for the maintenance of natural ecosystems (Pimentel, Zuniga, & Morrison, 2005).

Successful and rapid colonization of novel habitats by non‐native species often occurs despite genetic limitations stemming from founder effects that are typically associated with extinction risk (e.g., low diversity and inbreeding depression; Frankham, 2005). This “genetic paradox” has largely been resolved through meta‐analyses that have identified key components that lead to successful establishment of newly founded populations. In particular, studies have shown that large propagule size and multiple introduction events (i.e., propagule pressure) allow species to maintain and sometimes surpass levels of neutral genetic diversity relative to native sources and expand their introduced range (Lockwood, Cassey, & Blackburn, 2005; Roman & Darling, 2007; Consuegra, Phillips, Gajardo, & de Leaniz, 2011; Kolbe, Leal, Schoener, Spiller, & Losos, 2012). Admixture of genetic lineages within species and introgressive hybridization among species can also increase genetic diversity and produce novel genotypes (e.g., Kelly, Muirhead, Heath, & MacIsaac, 2006). Maintenance of genetic variation is expected to allow for selection to occur in introduced species, and several studies have demonstrated that adaptive genetic change can occur rapidly (Huey, Gilchrist, Carlson, Berrigan, & Serra, 2005; Stockwell et al., 2003; Kinnison, Unwin, & Quinn, 2008). Thus, genetic variation can play a complex role in the establishment of introduced species in new environments (Dlugosch, Anderson, Braasch, Cang, & Gillette, 2015; Roman & Darling, 2007).

In addition to genetic variation, several factors influence the establishment success of organisms including environmental similarity between source and founding locations, life history variation, biotic interactions, and demographics (Arismendi et al., 2014; Bock et al., 2015; Facon et al., 2006). Species that are introduced to areas with similar climate and habitat as their source locations often have high establishment success (Hayes & Barry, 2008; Moyle & Marchetti, 2006), but some species become established in environments that differ from their native range through life history variation (Facon et al., 2006; Sax et al., 2007). Species that become established in dissimilar habitats typically display broad phenotypic plasticity and environmental tolerance (Arismendi et al., 2014) and may experience fewer competitive and biological pressures compared to their source niche (Keane & Crawley, 2002). Demographic factors such as dispersal (natural or human‐mediated) contribute to initial colonization of non‐native species, while connectivity of established populations may facilitate maintenance and expansion of invasive species (Facon et al., 2006).

Despite the many factors that must be considered for studies of the invasiveness of non‐native species, new molecular tools offer the potential to address both demographic and evolutionary processes by surveying neutral and adaptive genetic variation throughout the genome (e.g., Davey et al., 2011; Narum, Buerkle, Davey, Miller, & Hohenlohe, 2013). Neutral loci are effective for evaluating demographic factors such as genetic diversity and gene flow, while adaptive variation can reveal signals of selection when introduced collections are compared to those in a species’ native range (e.g., Hamilton, Okada, Korves, & Schmitt, 2015). Further, species that are introduced to novel geographic regions without native congeners provide effective study systems to investigate invasion genetics as there is no confounding genetic background from extant populations or closely related taxa.

This study examines two salmonid species with differing life histories and invasion success after introduction to new environments in Patagonia, South America. In South America, salmonids (Salmonidae) were not historically native but have been widely introduced to rivers and lakes throughout Patagonia with similar environments as their native ranges (MacCrimmon, 1971; Pascual et al., 2007; Basulto, 2003; Gallardo et al., 2007; Correa & Gross, 2008; Consuegra et al. 2011; Arismendi et al., 2014; Monzón‐Argüello, de Leaniz, Gajardo, & Consuegra, 2014). Some species have been more successful than others at establishing viable populations in non‐native aquatic systems of South America, with 5 of 12 introduced salmonid species considered established (Arismendi et al., 2014; Monzón‐Argüello et al., 2014). Species such as Chinook Salmon (*Oncorhynchus tshawytscha*) are widespread and considered invasive, while others such as Brook Charr (*Salvelinus fontinalis*) are only locally established despite multiple introduction attempts for both species that were initiated in the early 1900′s, but more intensively in recent decades (Arismendi et al., 2014). In particular, Chinook Salmon aquaculture programs were initiated in the 1980's, and escapees from ocean net pens spread widely to rivers throughout Patagonia (Arismendi et al., 2014; Correa & Gross, 2008; Di Prinzio, Riva Rossi, Ciancio, Garza, & Casaux, 2015). Brook charr were heavily introduced for recreational fishing opportunities in various locations in Patagonia in the last 30 years, but there is little evidence that this species has become widespread (Arismendi et al., 2014; Gallardo et al., 2007). Here, we investigate Chinook Salmon and Brook Charr populations in South America, and contrast life history and genomic variation of introduced populations with stocks from their native range. Specific objectives included testing for the following: (i) patterns of genetic differentiation and connectivity within introduced populations relative to those from their native range, (ii) evidence of reduced genetic diversity in introduced populations due to founder effects, and (iii) evidence for divergent selection between native and introduced populations in differing environments.

## 2 Methods

### 2.1 Samples of introduced and native populations

**Figure 1:**
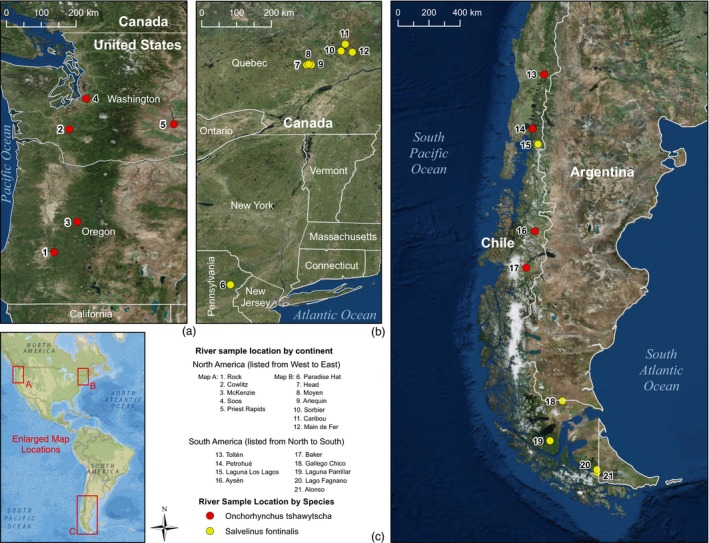
Map of sample collections for (a) Chinook Salmon in their native range from North America, (b) Brook Charr in their native range from North America, and (c) introduced populations of both species in South America

A major premise of our study design was to compare genomic variation of introduced populations in Patagonia, South America, to collections from their North America native ranges that represented putative source stocks as well as those under natural population dynamics. Samples from introduced populations in South America included four collections of Chinook Salmon and five collections of Brook Charr (Figure 1). As native ranges for each species are extensive spanning thousands of kilometers in North America, it was not feasible to compare native populations from across entire geographic distributions. Thus, we utilized information from previous studies to identify appropriate reference collections from native ranges to address study objectives. Specific stocks used for historical introductions to South America have not been thoroughly documented, but putative source stocks for each species have been identified in previous studies (Correa & Gross, 2008; Di Prinzio et al., 2015; Neville & Bernatchez, 2013) as described below.

Native Chinook Salmon collections were targeted based on information from a previous study (Correa & Moran, 2016) that identified the Pacific Northwest region of North America as the source of the specific introduced populations from Patagonia, South America, examined herein. Probable sources identified with microsatellite markers (Correa & Moran, 2016) included lower Columbia R. spring and fall runs, South Puget Sound fall run/Whidbey Basin, North Oregon Coast, and interior Columbia R (summer/fall run), which are all stocks from Oregon and Washington, USA. For this study, the five most representative native collections available were (i)—Cowlitz River (akin to lower Columbia R. spring run, Washington); (ii)—Soos Hatchery (akin to Puget Sound fall run, Washington); (iii)—Rock Creek (akin to north Oregon Coast); (iv)—McKenzie River (akin to north Oregon Coast); and (v)—Priest Rapids Hatchery (akin to interior Columbia R. fall/summer‐run, Washington; Figure 1a). These five native collections were included to test for contrasting patterns with the four introduced populations from South America for a total of nine Chinook Salmon collections (*n* = 364; Table 1).

**Table 1:** Genetic diversity and effective population sizes of collections of Chinook Salmon (*O. tshawytscha = Ots*; 11,759 SNP markers) and Brook Charr (*S. fontinalis = Sfo*; 13,522 SNP markers) from introduced (South America = S.A.) and native (North America = N.A.) locations. H_E_ = expected heterozygosity; AR = allelic richness; PAR = private allelic richness; N_e_ = effective population size with 95% confidence intervals (95% CI)

Species	Collection Description & Abbreviation	Sample size (*n*)	H_E_	AR	PAR	Nb^a^ (95% CI)
*Ots*	N.A.—Cowlitz River (COW)	45	0.337	1.21	0.024	927 (669–1498)
*Ots*	N.A.—McKenzie River (MCK)	48	0.337	1.20	0.026	442 (368–553)
*Ots*	N.A.—Priest Rapids Hatchery (PRH)	46	0.274	1.20	0.025	2471 (1234–315870)
*Ots*	N.A.—Rock Creek (ROC)	48	0.292	1.22	0.028	1243 (873–2144)
*Ots*	N.A.—Soos Creek (SOO)	48	0.265	1.21	0.032	1989 (1178–6265)
*Average across N. American populations (Ots)*	*47*	*0.301*	*1.21*	*0.027*	*1414*	
*Ots*	S.A.—Aysén Rio (AYS)	30	0.299	1.21	0.025	202 (179–233)
*Ots*	S.A.—Baker Rio (BAK)	34	0.353	1.21	0.023	356 (292–457)
*Ots*	S.A.—Petrohué Rio (PET)	36	0.305	1.22	0.027	67 (64–70)
*Ots*	S.A.—Toltén Rio (TOL)	19	0.283	1.20	0.031	149 (125–183)
*Average across S. American populations (Ots)*	*29.8*	*0.310*	*1.21*	*0.026*	*194*	
*Sfo*	N.A.—Arlequin Lac (ARL)	38	0.247	1.12	0.017	48 (45–51)
*Sfo*	N.A.—Caribou Lac (CAR)	30	0.316	1.17	0.01	16 (15–16)
*Sfo*	N.A.—Paradise Hatchery (PAR)	19	0.367	1.20	0.015	414 (250–1174)
*Sfo*	N.A.—Head Lac (HEA)	30	0.303	1.19	0.014	136 (123–152)
*Sfo*	N.A.—Main de Fer Lac (MDF)	29	0.224	1.12	0.012	461 (275–1366)
*Sfo*	N.A.—Moyen Lac (MOY)	29	0.226	1.08	0.013	133 (99–199)
*Sfo*	N.A.—Sorbier Lac (SOR)	27	0.333	1.18	0.009	199 (160–262)
*Average across N. American populations (Sfo)*	*28.9*	*0.288*	*1.15*	*0.013*	*201*	
*Sfo*	S.A. ‐ Alonso Rio (ALO)	39	0.299	1.23	0.019	101 (96–106)
*Sfo*	S.A. ‐ Gallego Chico Rio (CHI)	47	0.258	1.17	0.014	58 (56–60)
*Sfo*	S.A. ‐ Lago Fagnano (FGN)	15	0.305	1.23	0.018	601 (327–3441)
*Sfo*	S.A. ‐ Los Lagos Laguna (LAG)	41	0.289	1.17	0.029	233 (203–273)
*Sfo*	S.A. ‐ Parrillar Laguna (PAR)	41	0.222	1.17	0.014	112 (105–121)
*Average across S. American populations (Sfo)*	*36.6*	*0.275*	*1.19*	*0.019*	*221*	

a Nb estimates adjusted for downward bias following Waples et al. (2016).

Average values are shown in italics.

For Brook Charr, collections from the native range were targeted to include a hatchery stock (Paradise Hatchery, Pennsylvania, USA; Figure 1b) that has been commonly used for broad introductions over the past century (Neville & Bernatchez, 2013) and was expected to represent a likely source stock for introductions in Patagonia. However, this was not expected to account for all putative source stocks which were not feasible to include in this study. Additionally, six wild populations from the native range in Quebec, Canada, that have not experienced introgression with stocked fish from other regions (Marie, Bernatchez, & Garant, 2010) were included to represent genetic variation that would be expected in natural populations. Of the six wild populations, three were from the Portneuf system and three were from the Mastigouche system in Quebec, Canada. These seven native collections were included (Figure 1b) from North America along with the five introduced collections from South America for a total of 12 Brook Charr collections (*n* = 414; Table 1).

To contrast general environments of native versus introduced populations for each species, indicators of precipitation, temperature, and elevation were obtained for each sampling location from a database of global climate layers with high‐spatial resolution (WorldClim, www.worldclim.org; Hijmans, Cameron, Parra, Jones, & Jarvis, 2005). A total of 20 variables were extracted using ArcGIS software (ESRI; Redlands, CA, USA) including elevation and several bioclimatic indicators of temperature and precipitation (“bio01”—”bio19”; Table S1). These data were obtained from the “Current Conditions” dataset, which includes interpretations of observed data, representative of the years 1950–2000, at a resolution of 30 arc‐seconds (~1 km). Environmental relationships among collections were evaluated with principal component analysis (PCA) implemented in the R statistical package version 3.3.1 (R Development Core Team 2013). To aid interpretation of resulting canonical variables, vectors representing the original variables were passively correlated with the canonical axes and plotted onto the ordination using the “envfit” function of the R package “vegan” version 2.0‐10 (Oksanen et al., 2013).

### 2.2 Molecular techniques for RAD sequencing

DNA was extracted from fin tissue using Qiagen DNeasy kits (Qiagen) following the manufacturers’ recommended protocols. Extracted genomic DNA was quantified using Quantit PicoGreen dsDNA Assay Kits (Invitrogen) and a Victor2 microplate fluorometer (Perkin Elmer).

Restriction site‐associated DNA (RAD) libraries were prepared for Illumina HiSeq 1500 sequencing using a protocol similar to those previously published (Baird et al., 2008), but modified as described in Hecht, Matala, Hess, and Narum (2015). Libraries were prepared with a starting DNA concentration between 250 and 500 ng per sample, with samples of similar DNA quantity included in each library to provide equal representation of sequencing reads. Samples were digested individually with the restriction enzyme *SbfI*‐HF (NEB, Ipswich, Massachusetts, USA) and individually barcoded using a six nucleotide barcode adapter sequence. Digested and barcoded samples of the same starting concentration were pooled into libraries that averaged 48 individuals, where no two samples within a library were assigned the same barcode sequence, and each barcode sequence within a library differed by at least two bases from another barcode sequence. Libraries were then mechanically sheared using a Bioruptor 300 sonicator (Diagenode) to generate DNA fragment lengths between 200 and 700 bp, and fragments were isolated using an Agencourt AMPure XP bead purification system (Beckman Coulter). Prior to sequencing, RAD libraries were quantified using real‐time PCR on an ABI 7900HT Sequence Detection System (Life Technologies). Each library was sequenced in one lane on a HiSeq 1500 sequencer at a single read length of 100 bp, across a total of 16 lanes.

### 2.3 Bioinformatics pipeline and filters

SNP discovery was completed with the *de novo* pipeline in STACKS (Catchen, Amores, Hohenlohe, Cresko, & Postlethwait, 2011; Catchen, Hohenlohe, Bassham, Amores, & Cresko, 2013) with sequence data for each species. For Chinook Salmon, this included nine populations (five from North America and four from South America). For North America collections of Chinook Salmon, RAD data were available from a previous study (Hecht et al., 2015) with libraries prepared in an identical manner as those from South America and included 10 double haploid fish to test for paralogous sequence variants (PSVs). For Brook Charr, *de novo* SNP discovery included 12 populations (seven from North America and five from South America).

For the STACKS pipeline of each species, raw Illumina reads were first checked for quality using the program FastQC (http://www.bioinformatics.babraham.ac.uk/projects/fastqc/). The 25 bases on the 3′ end of sequence reads had reduced quality scores relative to the 5’ 75 base positions across our sequence data, so reads were trimmed to 75 bp. Reads were quality filtered and de‐multiplexed using the “process_radtags” program of STACKS, including options for cleaning the data by discarding any read with an uncalled base (‐c), discarding reads with low‐quality scores (‐q), and rescuing barcodes and partial restriction enzyme recognition sites (‐r). All other parameters and options were executed with the default values as outlined in the manual for the program (http://creskolab.uoregon.edu/stacks).

After individual sample reads were quality filtered, trimmed, and de‐multiplexed, sequences for each sample were taken through the “ustacks” module of STACKS to identify loci. In “ustacks,” the deleveraging (‐d) and removal (‐r) algorithms were applied to filter out those sequences that were likely to be paralogous and highly repetitive. Parameters for STACKS were minimum depth of coverage at a stack (“‐m” = 2–5), maximum distance between stacks (“‐M” = 2), distance between secondary reads, and primary stacks (“‐N” = 4). SNP discovery was carried out using the default SNP model with a chi‐square significance level of 0.05. We created a *de novo* catalog of RAD tag loci using the “cstacks” module by selecting two individuals from each population with at least 2.5 million reads (but no >4 million reads) to represent genetic variation in each species. Individual samples were then aligned to the catalog using the module “sstacks,” and genotypes were exported using the “populations” module.

Genotypes were filtered with multiple steps as shown in Table S2 including exclusion of: (i) any RAD tag locus with more than four SNP sites to remove putative PSVs, hypervariable, or poorly sequenced tags, (ii) any RAD tag locus where one of the ten double haploid samples was observed to be heterozygous at any of the SNP positions to remove putative PSVs, (iii) any SNP marker with more than two alleles to remove SNPs with sequencing errors, putative PSVs, or loci that do not fit a bi‐allelic statistical model, (iv) any SNP marker missing more than 30% of the genotypes across all of the populations to limit the amount of missing data, (v) any SNP marker failing tests of Hardy–Weinberg Equilibrium in more than one‐third of the populations to exclude technical artifacts such as null alleles (heterozygote deficit loci) and putative PSVs (heterozygote excess loci; GENEPOP v.4.0.6, Rousset 2008; false discovery rate corrected critical value BY‐FDR, Benjamini and Yekutieli 2001; Narum, 2006), and 6) any SNP marker with an average minor allele frequency (MAF) across the populations falling below 0.02 to exclude spurious rare SNPs or sequencing errors. Filters for loci that deviated from HWE were implemented to remove false positives due to PSVs or null alleles at the expense of possibly excluding some markers under selection (false negatives). As physically linked SNPs would bias population genetic estimates such as F_ST_, we only retained the first SNP marker per RAD tag. Individual samples were also filtered from the dataset if they were missing more than 30% of genotypes across all filtered loci.

Sequences from RAD tags for Chinook Salmon were aligned to those from a high‐density RAD‐based linkage map in this species (Brieuc, Waters, Seeb, & Naish, 2014) in an effort to determine the relative genetic position and linkage group assignment of loci. Alignment to the RAD database of Brieuc et al. (2014) was conducted using the end‐to‐end mode in the short sequence alignment software program *Bowtie2* v.2.2.3 (Langmead, Trapnell, Pop, & Salzberg, 2009) and with a threshold of mapping quality (MAPQ) score ≥ 2. Alignment of Brook Charr RAD tag sequences against a Brook Charr linkage map (Sutherland et al., 2016) resulted in low numbers of homologous markers, potentially due to the different library preparation method used for the map generation from that used in this study. Therefore, the MapComp approach (described in Sutherland et al., 2016) was applied to pair homologous as well as proximal anonymous markers with mapped markers using a reference genome as an intermediate (here the most recent version of the Atlantic Salmon reference genome, ICSASG_v2; GenBank AGKD00000000.4; Lien et al. 2016). The MapComp algorithm was applied with default settings, except that it was adapted to permit multiple anonymous markers pairing with a single mapped marker to obtain approximate genomic locations. Proximal markers were only considered when an estimated distance between markers on the reference genome was <1 Mb. All nonpositioned markers were considered as “unknown.”

### 2.4 Statistical analyses

Genetic diversity was estimated for all populations of each species with measures of expected heterozygosity (H_E_), allelic richness (AR), and private allelic richness (PAR) in HP‐RARE (Kalinowski, 2005). Differences in genetic diversity among collections were tested with analysis of variance (anova). Genetic relationships among populations within each species were estimated by pairwise F_ST_ (Weir & Cockerham, 1984).

To determine clusters of genetically similar collections, we used discriminant analysis of principal components (DAPC) from the “adegenet” package (v1.4‐2/ade4 v1.7‐2 Jombart, 2008; Jombart & Ahmed, 2011) in the R statistical computing environment (R Development Core Team 2013). The number of clusters was determined by running 10 iterations of the “find.clusters” module of “adegenet” for 1 to 12 possible cluster values (*K*). The Bayesian information criterion (BIC) values, which provide a metric of model selection similar to likelihood, were averaged across the 10 iterations and standard deviation was estimated for each value of K. The rate of change in BIC value was used to determine the most appropriate value of K (Evanno, Regnaut, & Goudet, 2005).

Estimates of contemporary effective population size (N_b;_ following Luikart, Ryman, Tallmon, Schwartz, & Allendorf, 2010) for collections of each species were generated using the linkage disequilibrium (LD) method implemented in the program N_e_ESTIMATOR (Do et al., 2014). The number of SNPs available for both species was computationally prohibitive for these analyses, so 10% of the markers were randomly chosen for Chinook Salmon and Brook Charr after removing any putative outlier loci (i.e., 1164 and 1339 SNPs, respectively). Since loci that are physically linked can bias estimates of effective size (Waples, 2006), we first used N_e_ESTIMATOR to detect pairs of loci that exhibited strong evidence of physical linkage (mean R^2^≥0.5) for more than a third of populations. Based on these criteria, we excluded one locus per pair that exhibited signs of physical linkage which resulted in the removal of eight loci for Chinook Salmon and 25 loci for Brook Charr. This revised dataset was used to generate estimates of N_b_ using the random mating model and exclusion of rare alleles (critical value *P*_*crit*_ = 0.05) following procedures described in previous studies (Candy, Campbell, Grinnell, Beacham, & Narum, 2015; Gruenthal et al., 2014). Additional estimates of N_b_ were generated with standardized sample sizes for Chinook Salmon and Brook Charr (i.e., *n* = 30 and 27, respectively) by randomly selecting individuals for analyses. These sample size criteria resulted in the exclusion of one Chinook Salmon population (i.e., Toltén) and two Brook Charr populations (i.e., Lago Fagnano and Paradise Hatchery) from analyses to estimate N_b_. Finally, we incorporated a correction for downward bias in estimates of “naïve” N_b_ (Waples, Larson, & Waples, 2016) using haploid chromosome number for each species (Chinook Salmon = 34; Brook Charr = 42).

Outlier tests were performed with PCAdapt (Duforet‐Frebourg, Bazin, & Blum, 2014), a Bayesian program based on a hierarchical factor model that jointly determines neutral structure and outlier loci using K latent factors. To identify candidate outlier loci, the model searches for markers that deviate from neutral expectations as measured by latent factors. This approach provides the ability to parse outlier loci into latent factors for specific population clusters rather than over all populations. This was a particularly important feature for our study as one of our objectives was to distinguish signals of selection in introduced populations rather than native populations. This method has been shown to have sufficient power and low false‐positive rates in independent evaluations of various outlier methods (Lotterhos & Whitlock, 2015). We ran PCAdapt with a burn‐in of 200 steps and 400 steps in the MCMC, with *K* = 4 for Chinook Salmon and *K* = 12 for Brook Charr. Values of K were determined from the mean squared error rate for each species following criteria from Evanno et al. (2005) and a range of K between 1 and 12. The SNP data were also scaled so that all markers had a variance equal to one in order to reduce false positives as previously recommended (Duforet‐Frebourg et al., 2014). For all loci considered to be candidates from PCAdapt, we used existing paired end data for each species to assemble longer sequence contigs from overlapping reads with the program Sequencher (v.5.3) to annotate potential genes. We attempted BLAST searches (match threshold of e^−10^; Blast2GO 3.1.3, Conesa et al., 2005) for outlier loci of each species using the contig sequences. For further annotation of candidates, we aligned contigs to the Atlantic Salmon genome (NCBI ICSAGS_v2) with Bowtie2 and searched for coding sequences within 5 kb of either direction of the genome (10‐kb window).

## 3 Results

### 3.1 Environmental differentiation between native and introduced locations

**Figure 2:**
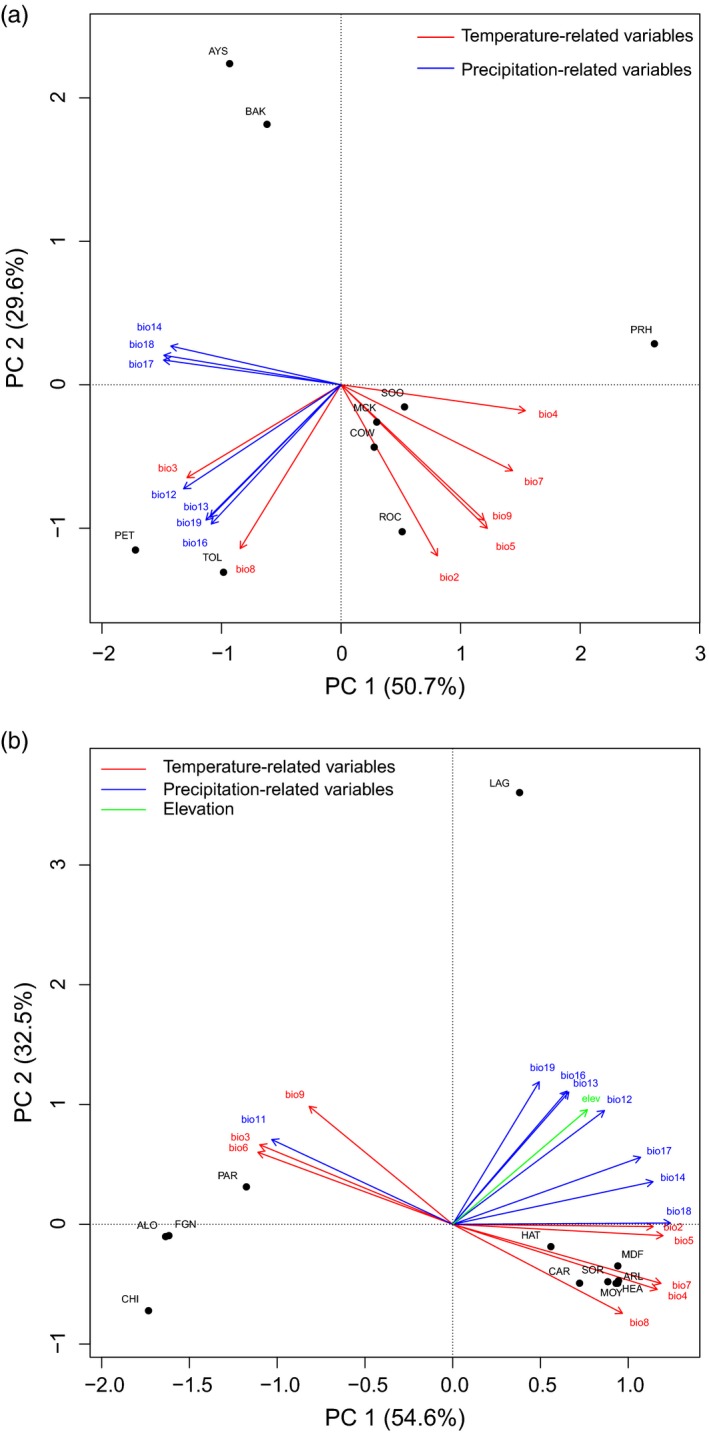
Principal component analysis (PCA) of environmental data for (a) Chinook Salmon, and (b) Brook Charr. Vector lines are only shown for environmental variables that accounted for a significant portion of the variation (*p *≤* *.01). Vector lines in red are temperature‐related variables, lines in blue are precipitation‐related variables, and elevation is green. Individual environmental variables are described in detail in Table S4, but “bio” codes of variables are as follows (WorldClim database): bio1 = Annual mean temp., bio2 = Mean diurnal range, bio3 = Isothermality, bio4 = Temperature seasonality, bio5 = Max temp. of warmest month, bio6 = Min temp. of coldest month, bio7 = Temp. annual range, bio8 = Mean temp. of wettest quarter, bio9 = Mean temp. of driest quarter, bio10 = Mean temp. of warmest quarter, bio11 = Mean temp. of coldest quarter, bio12 = Annual precipitation, bio13 = Precipitation of wettest month, bio14 = Precipitation of driest month, bio15 = Precipitation seasonality, bio16 = Precipitation of wettest quarter, bio17 = Precipitation of driest quarter, bio18 = Precipitation of warmest quarter, elev = elevation. Population abbreviations follow those in Table 1

The PCA of environmental features for both species indicated that the introduced locations in South America were distinct from those in North America and typically most similar within each continent (i.e., the first two components explained ≥80% of the variation for each species; Figure 2a,b). For Chinook Salmon, 13 of the 20 variables were statistically different among locations (*p *≤* *.01; Table S3a) and environmental differences between N. and South American locations were primarily driven by higher precipitation and cooler temperatures in the South American collections. Also for Chinook Salmon, there was distinction on PC2 among environments within South America between northern collections from Toltén and Petrohué relative to those from the south (i.e., Aysén and Baker) primarily due to lower precipitation and colder temperatures in the two southern locations. In North America, the Chinook Salmon collection from Priest Rapids in the interior was distinct on PC1 from all the other collections on the coast due to warm and dry conditions and more variable seasonal temperatures. For Brook Charr, 17 of the 20 variables were significantly different (*p *≤* *.01; Table S3b1) and collections were closely clustered by continent due to colder winter seasons with more precipitation in North America, with the exception of Los Lagos which was highly distinct in a more northern location with higher precipitation than all the other collections.

### 3.2 SNP Discovery, filters, and populations

#### 3.2.1 Chinook Salmon

*De novo* SNP discovery was completed for Chinook Salmon in STACKS with sequence data from nine populations (five from North America and four from South America). A total of 11,759 SNPs passed filtering criteria and were used for all further analyses. Table S4 includes the RAD tag sequence for all loci included for Chinook Salmon in this study. Of the 11,759 SNPs, 6,020 were aligned to an existing Chinook Salmon linkage map (Brieuc et al., 2014). Of the initial 364 samples from nine collections, 10 individuals were removed that did not satisfy criteria for missing genotype data (>30%). Mean sample size per population after filtering was 39.3 with a range of 19–48 per location (Table 1). Allele frequencies, individual genotypes, and HWE results are available in Table S5.

#### 3.2.2 Brook Charr

*De novo* SNP discovery was completed for Brook Charr with 12 collections representing seven native (North America) and five introduced (South America) populations. Our STACKS pipeline identified 13,522 SNPs that passed filtering criteria, of which 4,911 were assigned positions based on MapComp alignment with the Brook Charr linkage map (Sutherland et al., 2016). Table S6 includes the RAD tag sequence for all loci included for Brook Charr in this study. Of the initial 414 samples, 29 were removed that did not satisfy criteria for missing genotype data (>30%). Mean sample size per population after filtering was 32.1 with a range of 15–47 per location (Table 1). Allele frequencies, individual genotypes, and HWE results are provided in Table S7.

### 3.3 Genetic diversity and differentiation

#### 3.3.1 Chinook Salmon

No significant differences in genetic diversity were observed between native North America and introduced South America populations of Chinook Salmon (mean H_E_, South America = 0.310, North America = 0.301, *p *=* *.69; mean AR, South America = 1.21, North America = 1.21, *p *=* *.70; mean PAR, South America = 0.027, North America = 0.027, *p *=* *.70; Table 1). Despite similar levels of diversity, estimates of N_b_ revealed that introduced collections of Chinook Salmon had significantly lower N_b_ than native collections (mean N_b_, South America = 194, North America = 1414, *p *=* *.022; Table 1). These estimates of N_b_ were similar to those with standardized sample sizes (*n* = 30) among collections (mean N_b_, South America = 196, North America = 1257, *p *=* *.09; Table S1).

**Figure 3:**
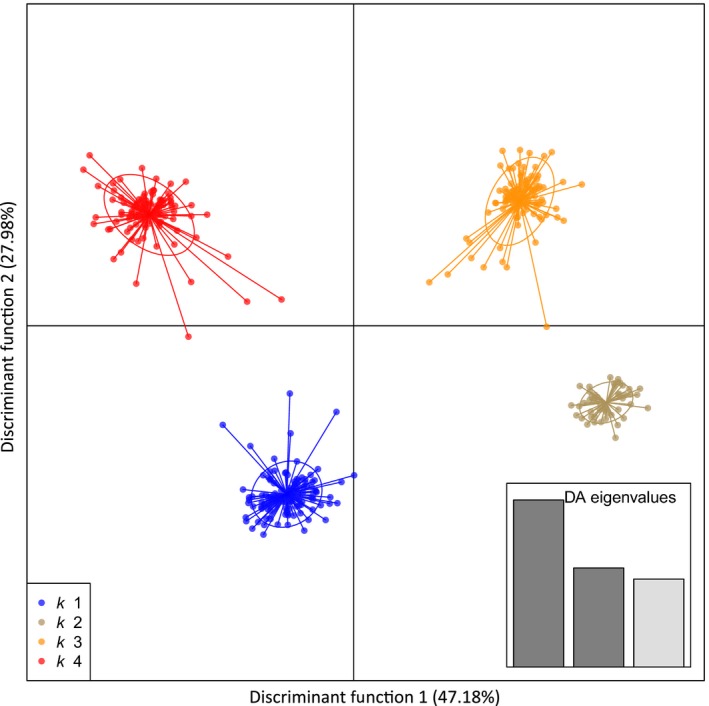
Discriminant analysis of principal components (DAPC) of Chinook Salmon (function 1 vs. 2) from 11,759 SNP markers. Cluster membership: k1 = Aysén, Baker, Cowlitz, k2 = McKenzie, k3 = Rock, Priest Rapids, k4 = Toltén, Petrohué, Soos

All populations of Chinook Salmon were significantly different from one another based on allele frequencies. Values of F_ST_ over all loci between North America and South America populations (mean = 0.074; range = 0.023–0.140) were similar to values among populations from the five native groups (mean = 0.088; range = 0.050–0.134), but F_ST_ values were generally lower among introduced populations (mean = 0.069; range = 0.011–0.106; Table S8). Relationships with DAPC identified four distinct clusters from the nine collections (*K* = 4) and explained much of the variation (DF1 = 47.2%, DF2 = 28.0%; Figure 3). The clusters from DAPC provided evidence for genetic similarity of Soos (North America) with Toltén, and Petrohué (South America) as one cluster, and Cowlitz (North America) with Aysén, and Baker (South America) as another cluster (Figure 3). Rock Cr. and Priest Rapids formed another cluster, while McKenzie R. was a single distinct cluster (Figure 3). Probability of individual membership strongly supported the four clusters as all but four individuals were assigned to their expected cluster with high probability (Fig. S1).

#### 3.3.2 Brook Charr

Estimates of genetic diversity were similar among populations of Brook Charr from South America and North America collections (mean H_E_, South America = 0.275, North America = 0.288; mean AR, South America = 1.19, North America = 1.15; mean PAR, South America = 0.019, North America = 0.013; Table 1). While South America populations did not have significantly different H_E_ (*p *=* *.65) or AR (*p *=* *.10) than those from North America, PAR (*p *=* *.04) was statistically significant. The difference in private alleles for Brook Charr was largely driven by higher PAR (0.029) in the Los Lagos collection relative to all other collections (South America range from 0.014 to 0.019; North America range from 0.010 to 0.017; Table 1). When Los Lagos was excluded, there were no significant differences in PAR between native North America and introduced South America populations (*p *=* *.07). Estimates of N_b_ revealed that both native and introduced collections of Brook Charr had low N_b_ that were not significantly different with all individuals included (mean N_b_, South America = 221, North America = 201, *p *=* *.659; Table 1) or with standardized sample sizes (*n* = 27) among collections (mean N_b_, South America = 129, North America = 162, *p *=* *.659; Table S1).

**Figure 4:**
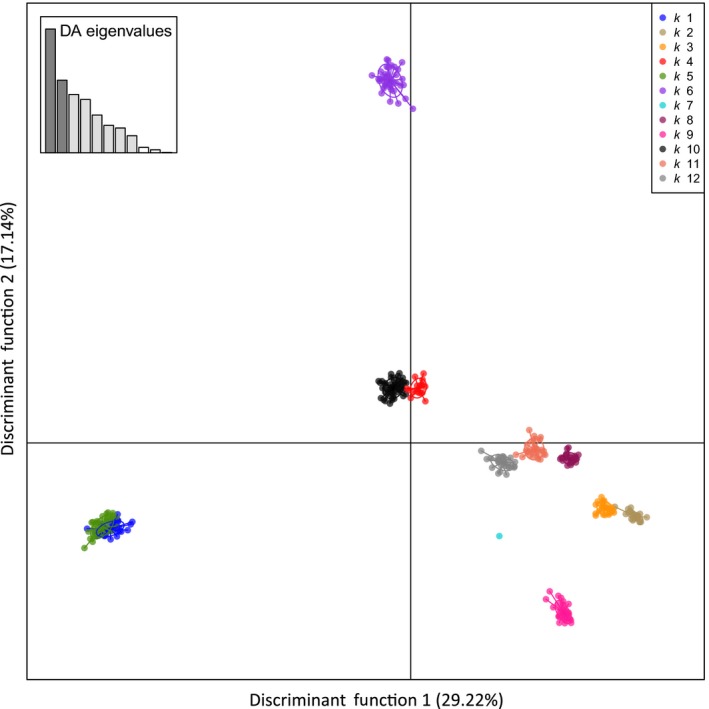
Discriminant analysis of principal components (DAPC) of Brook Charr (function 1 vs. 2) from 13,522 SNP markers. Primary cluster membership: k1 = Chico (South America), k2 = Main de Fer (North America), k3 = Sorbier (North America), k4 = Paradise (North America), k5 = Parrillar (South America), k6 = Los Lagos (South America), k7 = Arlequin (North America), k8 = Moyen (North America), k9 = Arlequin (North America), k10 = Alonso and Fagnano (South America), k11 = Head (North America), k12 = Caribou (North America)

All populations of Brook Charr were significantly different from one another based on allele frequencies with the exception of Lago Fagnano and Alonso River that were from the same drainage in South America (Alonso River is a tributary to Lago Fagnano). High values of F_ST_ over all loci indicated strong differentiation among Brook Charr collections. Values of F_ST_ between North America and South America populations (mean = 0.263; range = 0.070–0.429) were similar to values among populations from North America locations (mean = 0.259; range = 0.113–0.440), but F_ST_ values were generally much lower among introduced populations in South America (mean = 0.133; range = 0.013–0.243; Table S9). Mean F_ST_ among South America collections remained low (0.146) even when the nonsignificant comparison between Lago Fagnano and Alonso River was removed. The signal of strong genetic differentiation among Brook Charr collections was also apparent from DAPC results (DF1 = 29.2%, DF2 = 17.1%). Nearly all collections were distinct (*K* = 12), with the exception of Lago Fagnano and Alonso River from the same system that formed a single cluster that was most similar to the putative source stock from Paradise Hatchery (Figure 4). The other three introduced collections from South America were highly distinct from any North America collections included in this study. Probability of individual membership strongly supported the 12 clusters with only two individuals mis‐assigned at probability >0.90; however, assignment probability was generally lower for individuals between Parrillar Laguna and Gallego Chico clusters relative to other groups (Fig. S2).

### 3.4 Outlier tests for divergent selection

#### 3.4.1 Chinook Salmon

**Figure 5:**
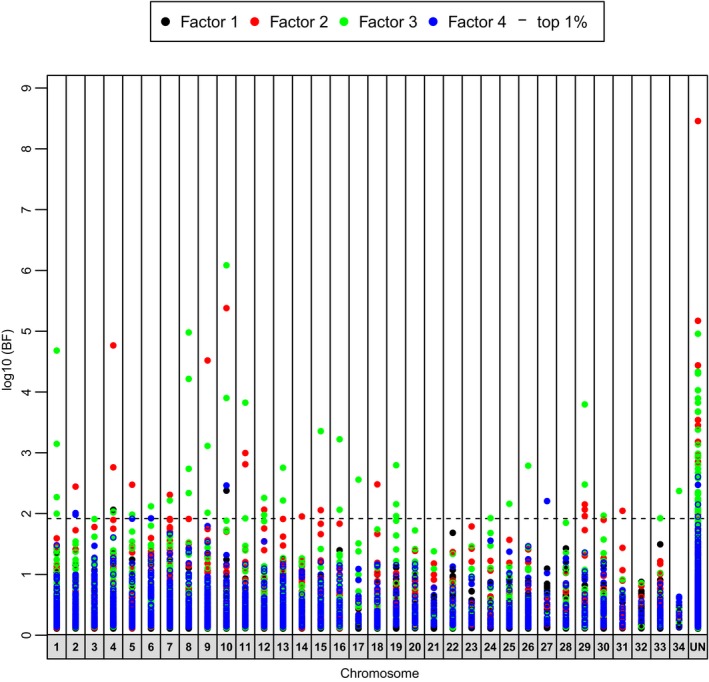
Outlier loci for each factor from PCAdapt analyses for Chinook Salmon with 11,759 SNP markers. The top 1% loci are shown above the dotted line based on chromosome position (unmapped markers are in the “UN” bin). Population abbreviations follow those in Table 1. Factors are represented by the following: F1 = Toltén/Petrohué from native, F2 = exotic from native, F3 = Toltén/Rock from all others, F4 = exotic from native

Tests for outlier loci were completed with PCAdapt to jointly account for underlying neutral structure and divergent selection. For Chinook Salmon, PCAdapt identified four significant latent factors (*k* = 4) among the nine collections. Differentiation among populations was evident for specific latent factors (Factor 1 = Toltén/Petrohué/Soos from others, Factor 2 = Soos/McKenzie versus Priest, Factor 3 = Toltén/Rock from all others, Factor 4 = introduced+Cowlitz from others; Figure 5). Latent factors explained variation that allowed examination of outlier loci that were associated with specific population groups (Factor 1 versus Factor 3, Fig. S3a; Factor 2 versus Factor 4, Fig. S3b). Of the top 1% outlier loci (Figure 5; Table S10), the majority were evident for Factor 3 (66.9%) and Factor 2 (23.7%), but a small proportion were also included for Factor 4 (7.6%) and Factor 1 (1.7%). These results suggest the occurrence of adaptive variation in introduced populations to South America, particularly in the Toltén River—which was specifically accounted for in Factor 3. Examination of allele frequencies of the 117 candidate loci for Chinook Salmon (Table S10) indicated that none of the outliers were fixed for alternative alleles among populations, and all outlier loci were polymorphic in one or more population from the native range. We were able to align paired end sequences for 111 of 117 outlier loci to achieve longer search strings (mean = 444 bp; Table S10) for BLAST searches and alignment to the Atlantic Salmon genome. With these 111 loci, 47 aligned in the BLAST search at the e^−10^ threshold. Several gene functions were represented in the BLAST hits and include several pathways (Table S10). Of the 111 sequences, we also aligned 85 of these to the Atlantic Salmon genome with MAPQ scores ≥3 (Table S10).

#### 3.4.2 Brook Charr

**Figure 6:**
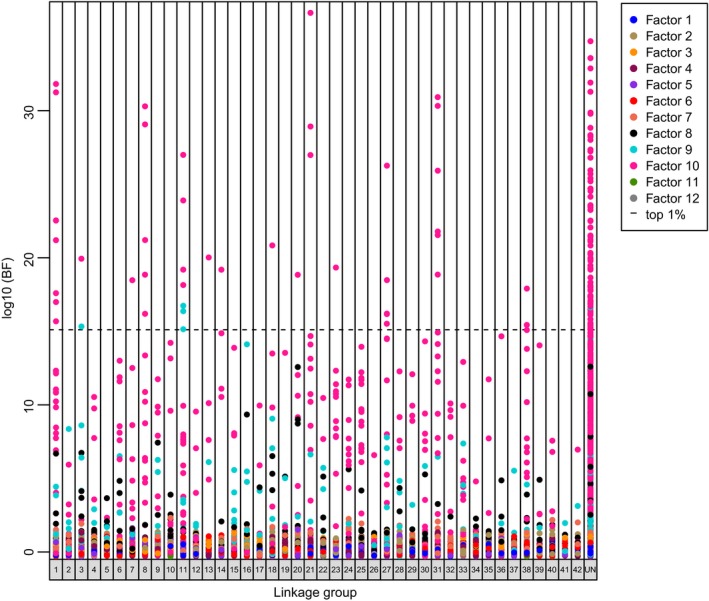
Outlier loci for each factor from PCAdapt analyses for Brook Charr with 13,522 SNP markers. The top 1% loci are shown above the dotted line and are represented by Factor 9 (Paradise Hat from all other populations) and Factor 10 (Parrillar and Gallego Chico from all other populations). Population abbreviations follow those in Table 1

Outlier tests for Brook Charr in PCAdapt were completed with 12 latent factors (*K* = 12) among the 12 collections. Differentiation among populations for each factor is shown in Figure 6, but our results focused on only two factors (Factor 9 and Factor 10; Fig. S4) that were represented in the top 1% of outlier loci (Table S11). Of the top outlier loci, 96.3% were associated with Factor 10 that represented Parrillar and Gallego Chico populations relative to all other populations, while the remaining 3.7% were associated with fish from Paradise Hatchery relative to all others in the study (Factor 9). These results may support adaptive hypotheses in these collections. Examination of allele frequencies of the 135 candidate loci for Brook Charr (Table S11) indicated that most outliers (74.8%) were due to differences in allele frequencies among populations rather than fixed differences. However, some outlier loci (34 of the 135) that were polymorphic in introduced populations were fixed in populations that were included from the native range. We were able to align paired end sequences for 129 of 135 outlier loci to achieve longer search strings (mean = 477 bp; Table S11) for BLAST searches and alignment to the Atlantic Salmon genome. With these 129 loci, 51 aligned in the BLAST search at the e^−10^ threshold (Table S11). Of the 129 sequences, we also aligned 108 of these to the Atlantic Salmon genome with MAPQ scores ≥3 (Table S11).

## 4 Discussion

Genomic variation plays a potentially complicated role in establishment of introduced species to new environments (Dlugosch et al., 2015). Here, genomic variation of introduced Chinook Salmon and Brook Charr in South America revealed patterns of diversity, population structure, and selection that may be related to invasion success. However, differences in dispersal potential between these two species appeared to be a driving factor of genomic variation and extent of their distribution. Specifically, introduced Chinook Salmon appear to have dispersed broadly in Patagonia by colonizing new freshwater rivers through anadromous migration or escaping from ocean‐rearing pens (Correa & Gross, 2008; Di Prinzio et al., 2015), while resident Brook Charr remain relatively isolated in drainages where they were physically stocked by humans with little evidence for natural colonization of new systems (Arismendi et al., 2014; Gallardo et al., 2007). The intensity of stocking efforts likely contributed to the current distribution of each species, but life history diversity and potential for migratory dispersal appear to have been key factors in range expansion.

Each of the introduced species had putative source stocks and natural populations from their native range that provided important references for comparison with introduced populations in South America. For Chinook Salmon, collections from Aysén and Baker rivers in Patagonia clustered with the Cowlitz collection from the lower Columbia River in North America, and the Toltén and Petrohué collections from Patagonia were most similar to the source of Soos Hatchery from Puget Sound in North America. These genetic stocks correspond to a large extent to specific source populations that have been identified in a previous study with very dense representation of source collections (146 native populations from 46 genetic lineages; Correa & Moran, 2016). The other three collections of Chinook Salmon from North America allowed us to account for patterns of natural genomic variation as well as scenarios of colonization from multiple sources or possible admixture (Correa & Gross, 2008; Di Prinzio et al., 2015). Despite the first introduction attempts in the early 1900's, stocking history and genetic analyses indicate that current populations of Chinook Salmon originated from introductions in Chile for ocean ranching in the late 1970s and early 1980s primarily from the lower Columbia River (Cowlitz River Hatchery spring‐run; Correa & Gross, 2008; Di Prinzio et al., 2015). In fact, this lineage prevails in our samples south of 43°S (i.e., Aysén and Baker; Correa & Moran, 2016). Additional accidental escapes from net‐pen aquaculture in the 1990s explain a more diverse assembly of lineages in the north (i.e., Toltén and Petrohué) traceable to South Puget Sound (akin to Soos Hatchery), interior Columbia Basin (akin to Hanford Reach and Priest Rapids), and other sources (Correa & Moran, 2016).

For Brook Charr, Paradise Hatchery from North America was a probable source stock representing Lago Fagnano and Alonso River in the extreme southern end of Patagonia. This was consistent with expectations that Paradise Hatchery would be a possible source stock as it has been identified as a common source for broad introductions in other geographic regions outside the species’ range (Neville & Bernatchez, 2013). The other collections of Brook Charr from Patagonia were highly distinct from those in the native range in Quebec, Canada, which is consistent with the high level of genetic divergence commonly observed in this species (e.g., Marie et al., 2010). Results also indicated that additional unidentified source stocks were used for introductions and likely contribute to signals of genetic structure of Brook Charr in Patagonia. Although Brook Charr introductions were attempted in Patagonia in the early 1900's (Arismendi et al., 2014), stocking records of Brook Charr show that there was only one documented introduction event in 1950 to Laguna Parrillar and Gallego Chico R., but multiple events since 1977 for Lago Fagnano, and between 1931 and 1971 for Los Lagos (Gallardo et al., 2007; Macchi, 2004). Records also indicate that fish were derived from non‐native stocks reared in Argentina that had been previously introduced in waters in southeastern Patagonia (Gallardo et al., 2007).

Levels of genetic diversity in the introduced populations of each species were not significantly different than estimates of diversity from native stocks, which reflected evidence of propagule pressure such as multiple introduction attempts and admixture of source stocks. This is consistent with reviews of studies of invasive species that genetic bottlenecks in founding populations are often overcome by propagule pressure (Dlugosch et al., 2015; Lockwood et al., 2005; Roman & Darling, 2007). In Chinook Salmon, genetic diversity of Patagonia stocks has probably been maintained by admixture or multiple colonization events from various stocks that escaped from ocean ranching (Di Prinzio et al., 2015). Given the significantly lower N_b_ of Chinook Salmon that we observed in South America collections relative to native stocks, ongoing colonization events and admixture would explain how genetic diversity has been maintained. Admixture and colonization could have caused a downward bias in our estimates of N_b_ for Patagonia stocks, but these factors would not have a large effect on estimates unless migration is high (i.e., 10 times the equilibrium rate; Waples & England, 2011).

In contrast, limited evidence for genetic bottlenecks in introduced Brook Charr relative to native stocks was rather surprising as this resident species is typically isolated and has had generally lower propagule pressure than many other introduced salmonids in South America (Consuegra et al. 2011; Arismendi et al., 2014). Stocking frequency may have an effect though as the two Brook Charr populations that were founded from a single stocking event (Parillar and Gallego Chico) had lower diversity and effective sizes than other introduced populations with repeated stocking events. This suggests that even relatively low‐intensity propagule pressure may be sufficient to retain genetic diversity for populations that were founded from stocks with low diversity and small effective size. However, examination of additional populations and more information regarding stocking history would be necessary to confirm this hypothesis.

Patterns of genetic structure differed between species introduced to Patagonia, with relatively low genetic differentiation for widespread Chinook Salmon compared to localized populations of Brook Charr with high divergence. These patterns of genetic structure are consistent with the hypothesis that distinct introductions coupled with connectivity and gene flow may be important for establishing populations on a broad geographic scale, consistent with reviews of introduced salmonids (Arismendi et al., 2014) and other invasive aquatic species (Roman & Darling, 2007). Once released or escaped from ocean aquaculture pens, anadromous Chinook Salmon have opportunities for colonization of multiple river systems with suitable habitat (Di Prinzio et al., 2015). Also, these anadromous fish returning from the ocean to freshwater may not always be philopatric, and thus, colonization of new areas may occur (Keefer & Caudill, 2014; Quinn, 1993). This migratory characteristic may allow for existing populations to be bolstered by new migrants and persist through stochastic natural events that might otherwise lead to local extirpation. Diverse life histories for Chinook Salmon have been previously shown to be key factors for colonization success not only in Patagonia (Correa & Gross, 2008; Di Prinzio & Pascual, 2008), but also in other novel environments such as New Zealand (e.g., Quinn, Kinnison, & Unwin, 2001).

In contrast to high dispersal potential in Chinook Salmon, Brook Charr typically remain as residents in freshwater lakes and rivers, although anadromy is also observed in this species (Castric & Bernatchez, 2003; Dauwalter, McGurrin, Gallagher, & Hurley, 2014; Thériault, Bernatchez, & Dodson, 2007). Restricted migration tendency would reduce the potential for Brook Charr to disperse and colonize new areas, thus limiting connectivity of existing populations. In the native range of Brook Charr, differentiation is high and reflects isolation of these resident stocks with little potential for gene flow among populations (i.e., Castric & Bernatchez, 2003; Lamaze, Marie, Garant, & Bernatchez, 2012; Marie et al., 2010). In the introduced stocks, potential for gene flow through migratory dispersal is also limited as these are primarily resident populations, but there is likely gene flow occurring through repeated stocking events (Arismendi et al., 2014; Gallardo et al., 2007) as evidenced by estimates of genetic distance that were approximately twice lower than the native range. In their native range, Brook Charr are threatened by invasive rainbow trout (Larson & Moore, 1985; Rose, 1986) which suggests that biotic resistance is also a plausible scenario in Patagonia given the existence of other salmonids including resident rainbow and brown trout (Monzón‐Argüello et al., 2014).

Outlier loci were observed in both species, but only in specific collections and not for all introduced populations. Differences between native and introduced environments were observed for both species, but were within the variation that occurs across each species’ native range (Hecht et al., 2015; Lamaze et al., 2012), suggesting that selection pressure was not likely to be strong relative to genetic drift in the introduced environments. The outlier approach with PCAdapt (Duforet‐Frebourg et al., 2014) was chosen specifically for this study as it provided advantages over other popular methods (i.e., Lositan, Antao, Lopes, Lopes, Beja‐Pereira, & Luikart, 2008; Beaumont & Nichols, 1996; Foll & Gaggiotti 2008) such as the ability to parse outlier loci into latent factors. It is possible that both false positives and negatives persist in our outlier analyses (e.g., De Mita et al., 2013; Lotterhos & Whitlock, 2014; Narum & Hess, 2011). However, PCAdapt has been demonstrated to be a powerful approach to account for underlying neutral structure in independent simulations to reduce false positives (Lotterhos & Whitlock, 2015). Nevertheless, we interpret our results from PCAdapt with caution as false positives could have occurred due to limited representation of specific source populations and effects of admixture from multiple stocks.

In Chinook Salmon, the collection from Toltén R. had the most outlier loci compared to other introduced collections from South America. These outliers may be explained by unaccounted founder lineages as Toltén and Petrohué populations were founded by multiple sources (Correa & Moran, 2016), but largely concordant identification of source stocks suggests that the current study accounted for much of the genomic variation that contributed to these introduced populations. While multiple ancestry and admixture could affect the performance of outlier tests, this could also lead to enhanced standing genetic variation for selection to act upon in new environments. There was also evidence for selection in native populations such as the Rock and Priest Rapids collections, which have strong differences in migration distance and precipitation that have been found to be significant factors associated with local adaptation in the native range of Chinook Salmon (Hecht et al., 2015). Outlier loci included several markers that were aligned to genes related to immune function, and exposure to novel pathogens could be selective forces for species introduced to new environments (Monzón‐Argüello et al., 2014; Roman & Darling, 2007). However, many other pathways were represented by candidate loci and annotation with future resources may provide more extensive biological interpretation.

In Brook Charr, outlier loci were observed in the Parrillar and Gallego Chico populations but not in the other introduced collections. Comparisons to native populations in North America provided critical information regarding relative levels of genomic variation; however, only two of the five introduced Brook Charr collections (Lago Fagnano and Alonso) had a genetically similar native stock (Paradise Hat.) represented in our study. Lack of representative founder stocks for Parillar, Gallego Chico, and Los Lagos could have affected outlier results, but not consistently as no outliers were detected in the Los Lagos collection. False positives are known to occur in outlier tests due to genetic bottlenecks (Teshima, Coop, & Przeworski, 2006), but estimates of genetic diversity in the Parillar and Gallego Chico collections were similar to native collections with no significant evidence of bottlenecks. Annotation of outlier genes represented various pathways with no obvious biological signal, but many outliers remained unknown due to limited availability of genomic resources.

The results from this study are consistent with the notion that dispersal potential of aquatic species can influence the extent of invasiveness as long as surrounding environments provide suitable habitat and that genetic diversity may be retained even in species with relatively low propagule pressure and distinct population structure. This information is critical as invasive species become established and cause a myriad of negative impacts that can lead to extirpation or extinction for native species (Crawford & Muir, 2008; Taylor, Courtenay, & McCann, 1984; Townsend, 1996). In the case of salmonids that have been introduced to South America, impacts to native aquatic species have been extensive with severe reductions in indigenous fishes and other aquatic organisms (Arismendi et al., 2014; Correa & Hendry, 2012; Habit et al., 2010). Several species of salmonids are now pervasive in rivers and lakes of southern Chile and Argentina leading to altered ecosystem dynamics (Habit, Gonzalez, Ruzzante, & Walde, 2012), interference with trophic cascades (Elgueta, Gonzalez, Ruzzante, Walde, & Habit, 2013), and reduced native biodiversity (Arismendi et al., 2014; Correa, Bravo, & Hendry, 2012; McDowall, 2006; Vera‐Escalona, Habit, & Ruzzante 2015). Despite relatively low estimates of effective population size of invasive species, census sizes can be much higher (Kalinowski & Waples, 2002) and cause substantial impacts on native species.

Despite the negative consequences of introduced salmonids in South America, they continue to be promoted due to high economic value in fisheries and aquaculture production. Thus, there is a need to balance the social, economic, and ecological trade‐offs of these introduced species in South America (reviewed in Pascual et al., 2007). Studies that elucidate characteristics that contribute to invasion success and distribution of introduced species may help contribute to successful management scenarios (e.g., Harrisson, Pavlova, Telonis‐Scott, & Sunnucks, 2014). For example, introduced species such as Chinook Salmon with high propensity to disperse and colonize new areas are likely to impact native aquatic organisms across a broader region than species like Brook Charr that often remain isolated in the systems where they are planted. However, not all anadromous salmonids introduced to Patagonia have become widespread (Arismendi et al., 2014), which emphasizes the point that factors influencing colonization success can be highly complex (Dlugosch et al., 2015). Further studies that examine additional species of introduced salmonids are needed to clarify patterns of invasiveness in Patagonia.

## Data Sharing

Supplementary data of individual genotypes for all SNP loci are stored in a Dryad digital repository under accession ID: https://doi.org/10.5061/dryad.2f050
