# Supplementary figures and images for: Genomic patterns of diversity and divergence of two introduced salmonid species in Patagonia, South America
Source: Evol Appl. 2017 Mar 6;10(4):402–16. doi: 10.1111/eva.12464 (PMC5367078; doi:10.1111/eva.12464)

Figure S1

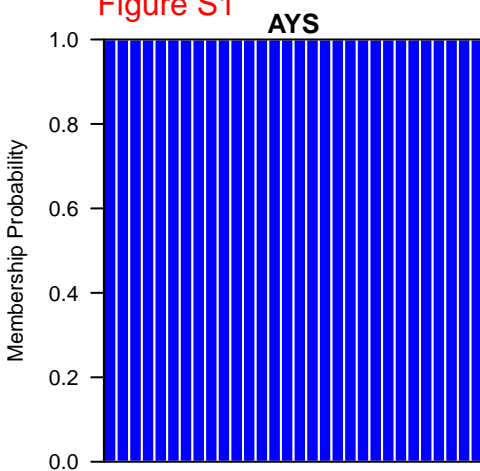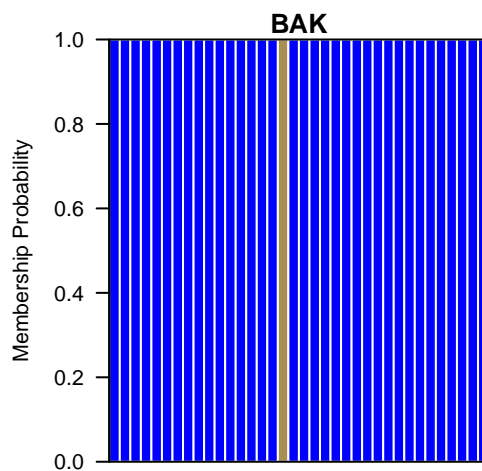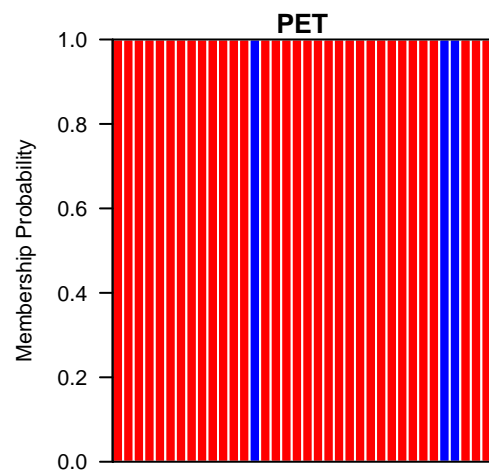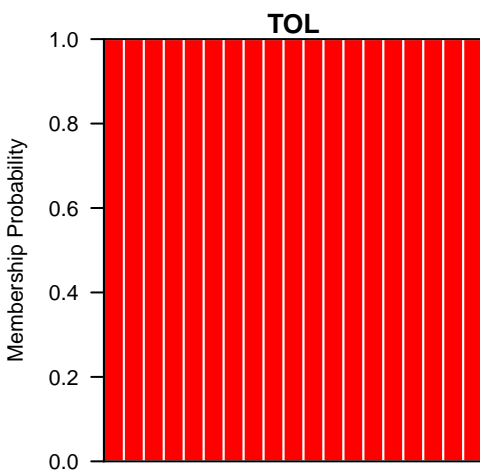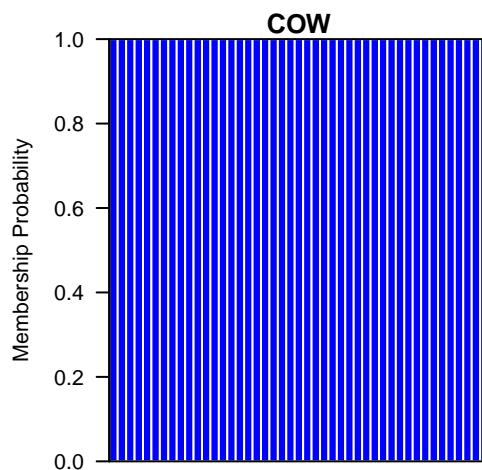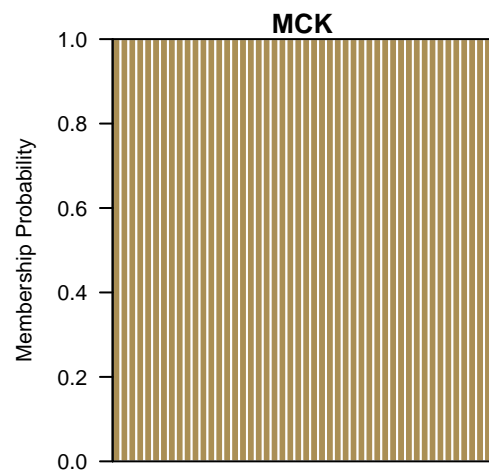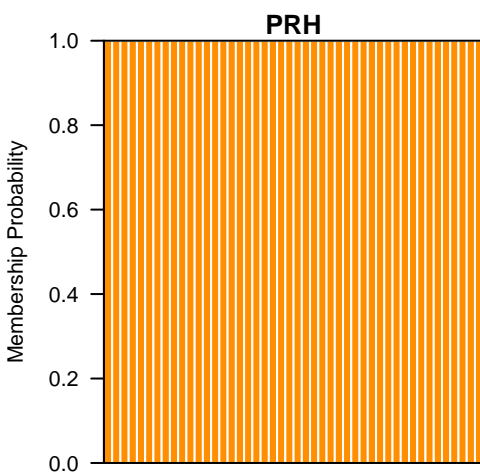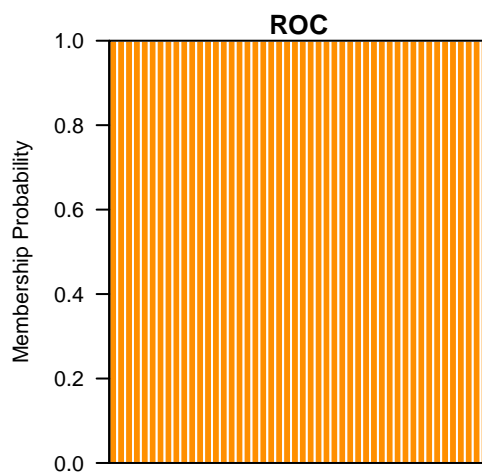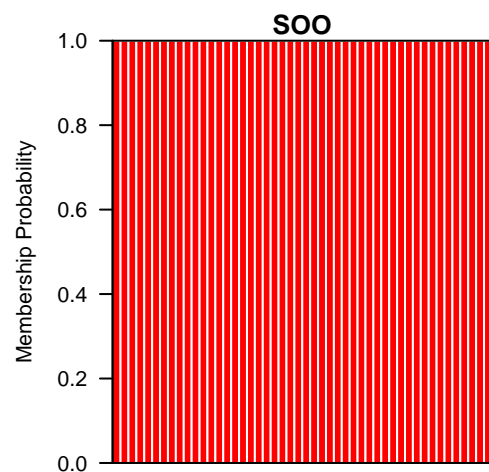

Figure S2

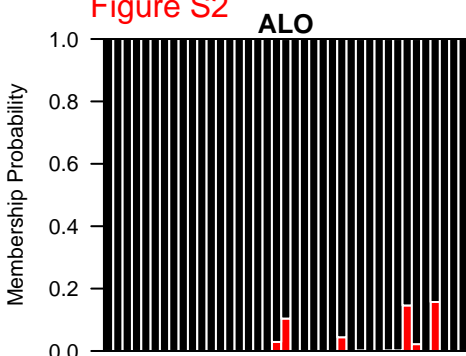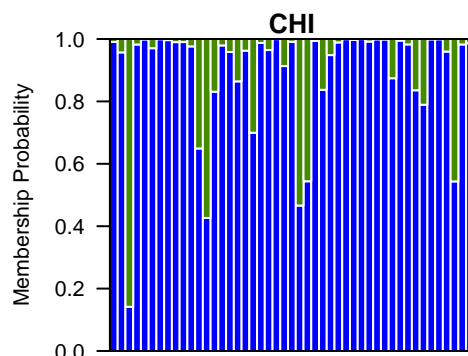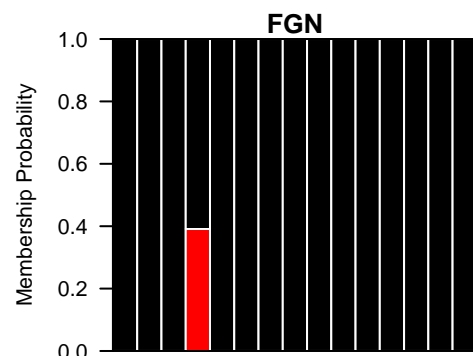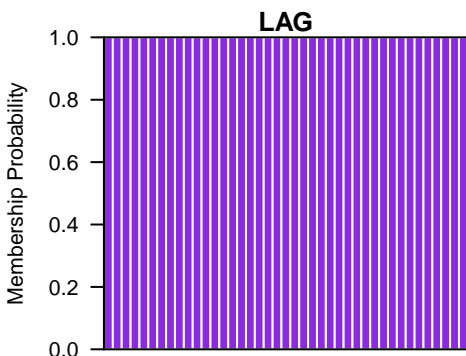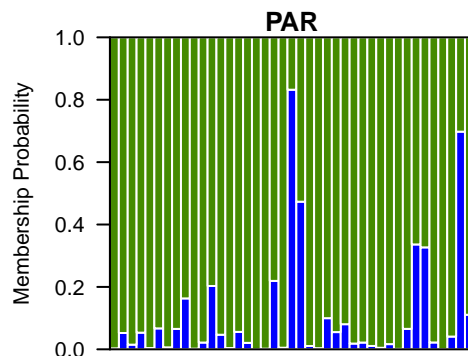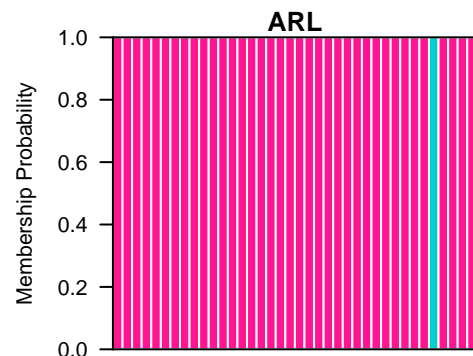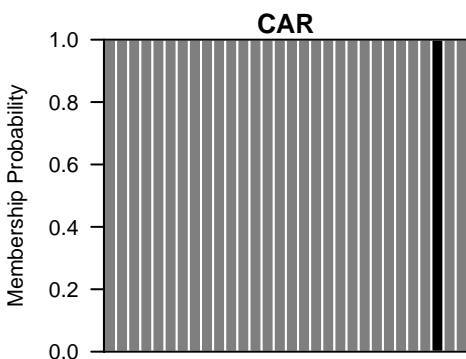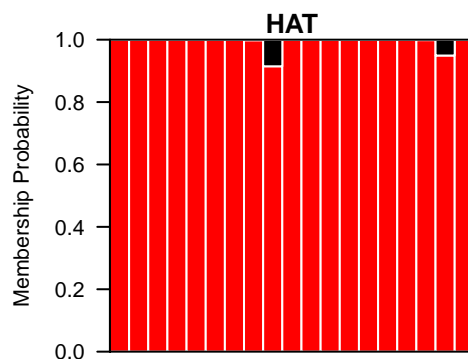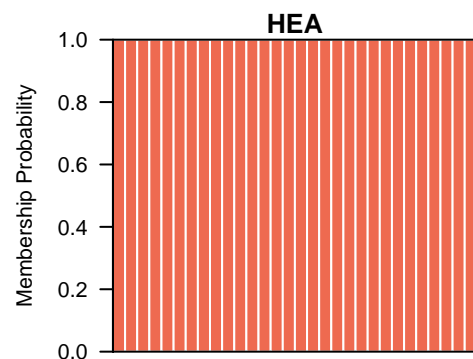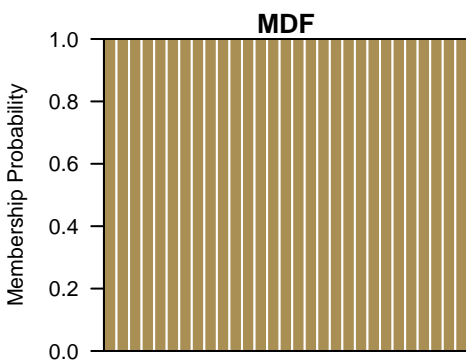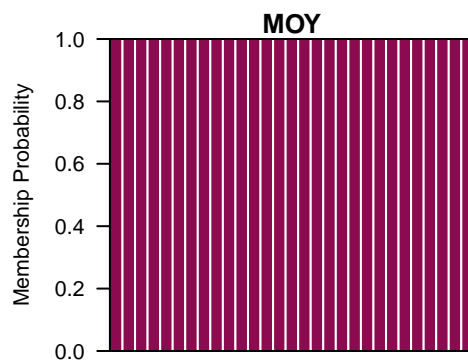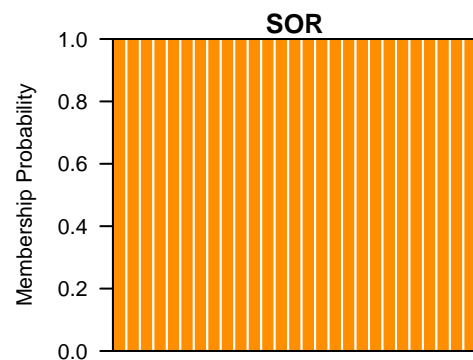

Figure S3a

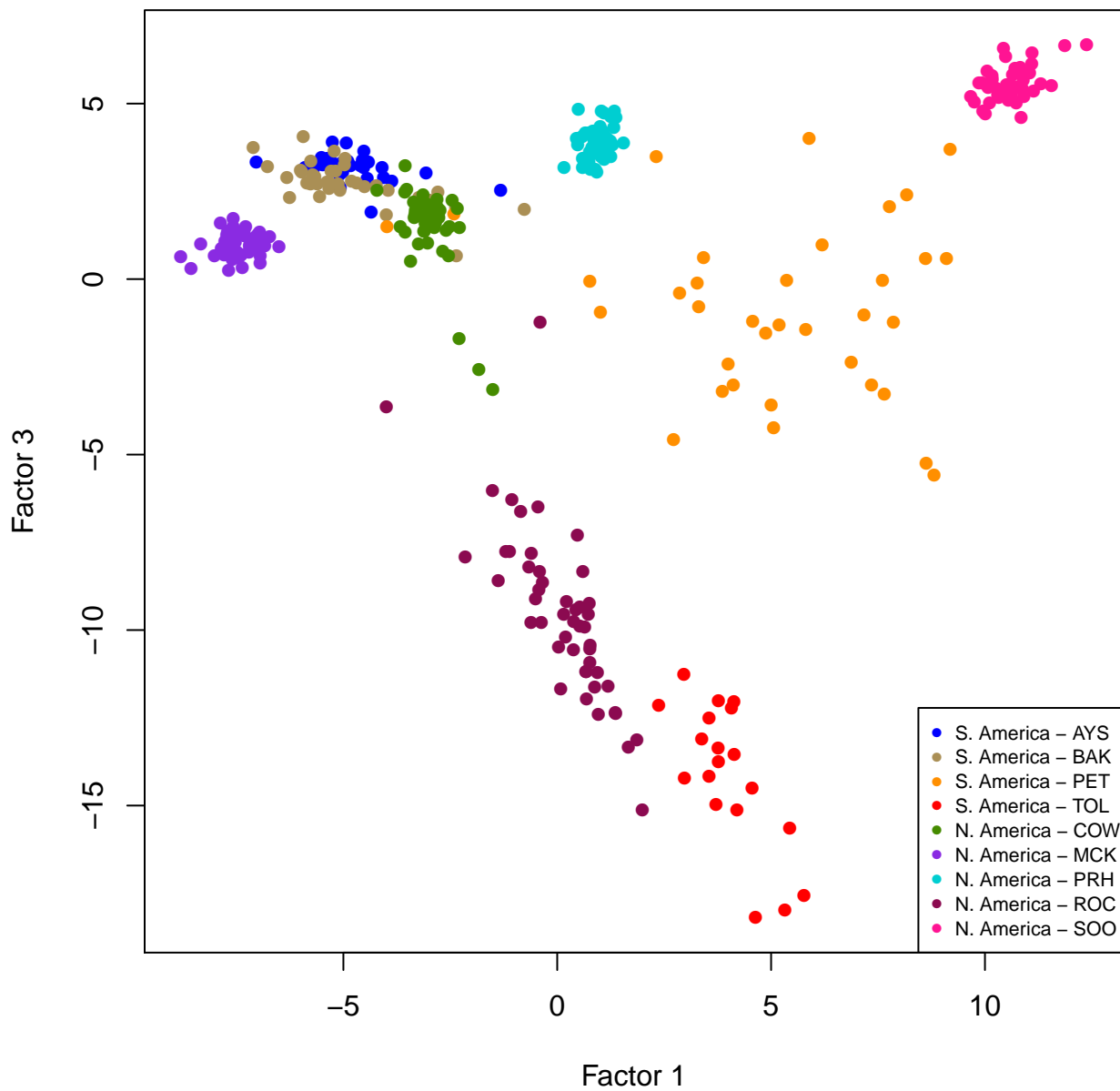

Figure S3b

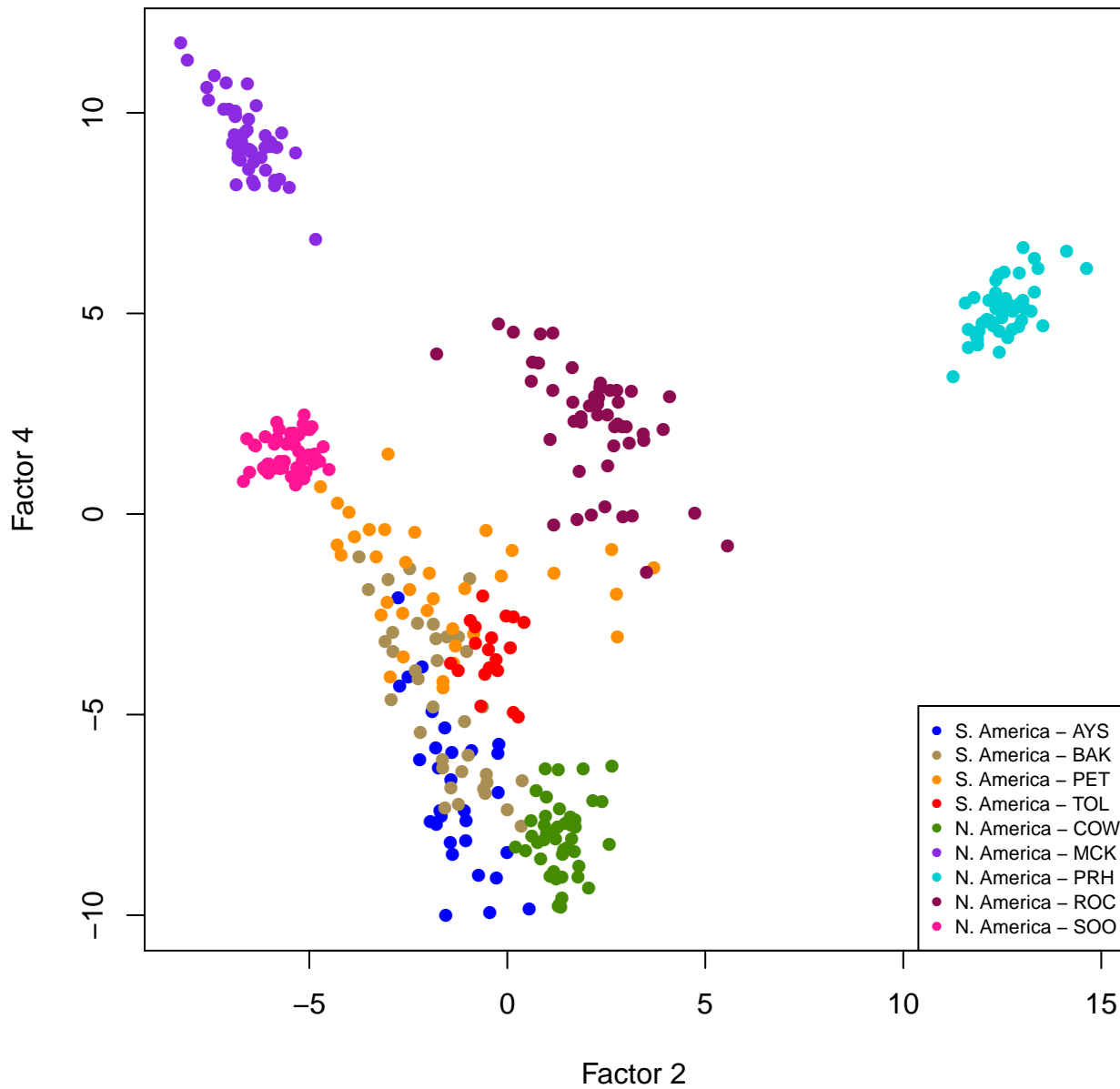

Figure S4

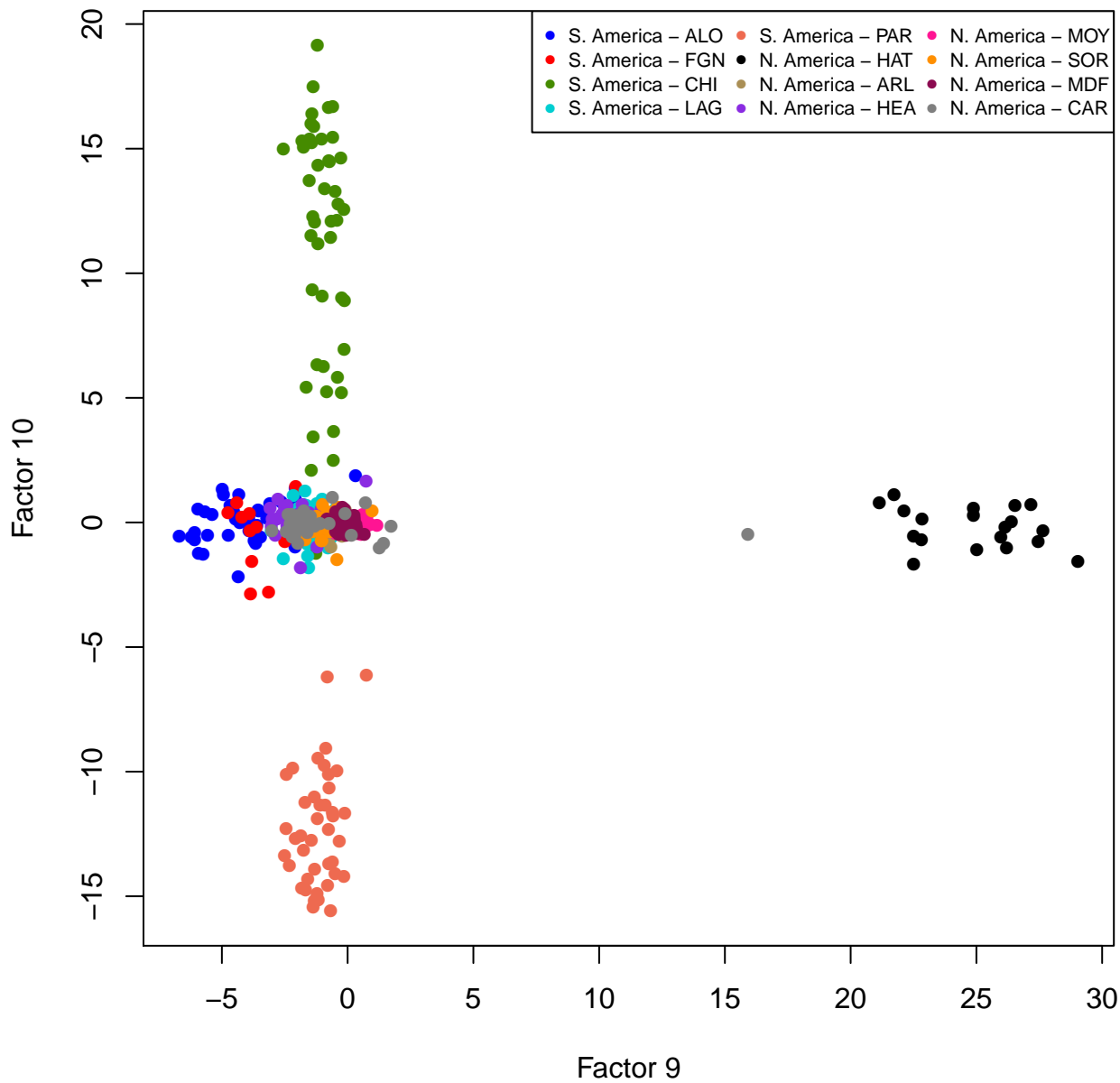

Supplement: Supplementary file 1 [file EVA-10-402-s001.pdf]
