# Supplementary material for: Genomic patterns of diversity and divergence of two introduced salmonid species in Patagonia, South America
Source: Evol Appl. 2017 Mar 6;10(4):402–16. doi: 10.1111/eva.12464 (PMC5367078; doi:10.1111/eva.12464)
Supplement: Supplementary file 3 [file EVA-10-402-s003.pdf]

Polyphyletic ancestry of expanding Patagonian Chinook salmon populations

AUTHORS: BLINDED DUE TO JOURNAL POLICY

Running title: Ancestry of Patagonian Chinook salmon

Number of words: 11063

Number of tables: 2

Number of figures: 3

Abstract

Viewed as great unplanned experiments, biological invasions can inform the potential for rapid adaptation in a changing world. Chinook salmon native to North America are spreading through South America's Patagonia and have become the most widespread anadromous salmon invasion ever documented (39-55°S). We tested whether this phenomenon could be explained by high adaptive potential related to the introduction of multiple distinct genetic lineages. Samples from four distant watersheds in Chile (39-46°S) were genotyped for 13 microsatellite loci, and allocated, through conditional maximum likelihood (CML) mixture analysis, to 148 potential donor populations in North America from 46 genetic lineages. Patagonian Chinook salmon had a diverse and heterogeneous ancestry. Lineages from the Lower Columbia River were introduced for salmon open-ocean ranching in the late 1970s and 1980s, and prevailed south of 43°S. In the north, however, a diverse assembly of lineages was found, associated with net-pen aquaculture during the 1990s. Simulations confirmed that CML mixture analysis is informative despite admixture, yet revealed misallocation of some hybrid genotypes to unrelated lineages. Patagonian Chinook salmon may exhibit rapid adaptive evolutionary responses due to artificially high levels of standing genetic variation, particularly in north-west Patagonia where multiple lineages exist in sympatry.

Key words: colonization; range expansion; invasive salmonids; admixture; assortative mating; anthropogenic secondary contact zone; aquaculture impacts;

Introduction

Multiple independent introduction events can lead to shifts in genetic variation relative to native source populations, potentially boosting invasiveness and potential for rapid local adaptation (Voisin et al. 2005; Roman 2006; Lucek et al. 2010; Rius and Darling 2014). Furthermore, different introduction vectors delivering distinct genetic lineages in different regions can result in a mosaic of populations with varying genetic diversity and evolutionary potential (Voisin et al. 2005; Roman 2006; Zalewski et al. 2010; Asif and Krug 2012). We investigated self-sustaining Chinook salmon [*Oncorhynchus tshawytscha* (Walbaum)] populations, currently part of an aggressive colonization that is sweeping through Patagonia, the binational region of Chile and Argentina at the southern cone of South America. Using microsatellite markers we detected strong population structure, identified multiple phylogenetic origins explained by artificial propagation programs of multiple Chinook salmon lineages in different regions, and found high population genetic diversity relative to native source populations. High genetic diversity of Patagonian Chinook salmon may confer high evolutionary potential for local adaptation, especially in north-western Patagonia where multiple lineages currently exist in sympatry.

Beginning in the 1870s, Chinook salmon, native to North America and the North Pacific Ocean, were deliberately introduced into innumerable rivers in all continents except Antarctica (Crawford and Muir 2007); yet successful naturalization has been rare. Self-sustaining adfluvial (migrating between lake and river) Chinook salmon populations have been established in the North American Great Lakes (Peck et al. 1999), but anadromous (migrating between the sea and river) populations outside their native range exist only in New Zealand's South Island and in South America's Patagonia (Crawford and Muir 2007). The phylogenetic ancestry of New Zealand Chinook salmon was tracked to introductions in the early 1900s from the Sacramento River fall run (seasons characterize

typical adult return to freshwater), most likely Battle Creek, California (McDowall 1994; Quinn et al. 1996, 2001). Originally stocked in one river of the South Island, the fish naturalized and within a decade expanded their range considerably (Quinn et al. 2001; Deans et al. 2004; Unwin 2006). When studied about 30 salmon generations later, a number of phenotypic traits had evolved apparently in response to local environmental conditions (Quinn et al. 2001; Kinnison et al. 2003), offering increased local fitness (Kinnison et al. 2008). The monophyletic ancestry and known history of introduction in New Zealand greatly facilitated that research (Quinn et al. 2001; Kinnison et al. 2008). It is noteworthy that measurable adaptive evolution seemingly took place despite monophyly and presumably limited founding genetic diversity available for natural selection to act upon. This rapid evolution in New Zealand underscores the potential of Chinook salmon to adapt to new environments, yet it begs the question of why successful introductions have been rare elsewhere.

Patagonian Chinook salmon are much less studied than New Zealand Chinook salmon. Patagonian populations are younger than those in New Zealand but much more widespread. Self-sustaining populations occur in a vast region on western slopes south of 38°S draining through Chile and into the Pacific Ocean. They are also established on eastern slopes south of 50°S draining through Argentina and into the Atlantic Ocean (Figure 1). The ancestral origin of these populations is unclear due to fragmentary historical records and insufficient study, and numerous potential donor populations must be considered (Becker et al. 2007; Astorga et al. 2008; Correa and Gross 2008; Riva-Rossi et al. 2012).

Correa and Gross (2008) reviewed the historical records of introductions into Latin America, records of salmon in the wild, locations of fish farming, and their own field observations. They concluded that, out of the many introduction attempts beginning in the late 1800s, two relatively recent experimental commercial open-ocean ranching operations were most likely the sources of the introduced populations. For the purpose of this study, we distinguish two classes of artificial

propagation. In open-ocean ranching, returning adults are spawned artificially in fish hatcheries and the juveniles are released to grow at sea. These free-ranging fish may be harvested at sea but especially in a terminal fishery of maturing adults homing back to the stream of release. The second class is floating net-pen aquaculture. In this case, fish are held captive from hatching until harvest or reproduction (see below). One open-ocean ranching operation took place on Chiloé Island (42°S) during the period 1978-1982 using primarily Cowlitz River Hatchery spring-run brood stock from the Lower Columbia River, Washington, U.S.A. (Lindbergh et al. 1981b). The other open-ocean ranching operation took place further south in the Magellan region (51°S) during the period 1982-1989 using University of Washington Hatchery fall-run broodstock, Puget Sound, Washington, as well as the progeny of adult fish returning to Chiloé Island (Donaldson and Joyner 1983; Méndez and Munita 1989).

Beginning in 1988, Chilean salmonid propagation shifted entirely from open-ocean ranching to net-pen aquaculture in captivity. Despite the captive design, fish escaped in the thousands every year and many of those escapes were poorly documented (Soto et al. 2001; Naylor et al. 2005; Buschmann et al. 2006; Arismendi et al. 2009). The stocks utilized, also poorly documented, were relatively diverse, as ova importations came at least from Washington, Oregon, Vancouver Island, and from New Zealand (the latter with ancestry from the Sacramento River, Central Valley, California) (Aedo 2011). In contrast to open-ocean ranching, net-pen aquaculture was mostly concentrated in Chile's Lake District region (42°S). A summary of Chinook salmon introductions to Patagonia is presented in Table 1. A complete review of introductions is provided as an online supplement (Table S1).

Comparative genetic analyses offer valuable insights into which introduced lineages naturalized and how they spread in Patagonia. Inferences are made based on genetic resemblance between Patagonian populations and reference (baseline) samples from a suite of potential donor population elsewhere. Becker *et al.* (2007) and Riva-Rossi *et al.* (2012) studied sequences within the

*mt*DNA control region, and compared Patagonian Chinook salmon to relatively few (<20) baseline populations from the native range and New Zealand. While Patagonian populations resembled some reference populations and not others, it was impossible to reliably determine the phylogenetic origin of Patagonian fish due to widespread sharing of *mt*DNA haplotypes in the native range of Chinook salmon, incomplete accounting of potential donor populations, and utilization of an haploid marker unsuited to detect admixture. More recently, Di Prinzio *et al.* (2015) and Ciancio *et al.* (2015) conducted a survey of nuclear markers (96 single nucleotide polymorphisms, SNPs), and included a wider range of baseline samples from 69 populations nested in 38 genetically identifiable lineages or reporting groups in the native range from Californa to Alaska (Clemento et al. 2014). With substantially improved resolution and discriminatory power, these studies suggested that Patagonian Chinook salmon can be traced to several North American source lineages, largely consistent with known introductions after 1979. Interestingly, a population on the Pacific slope (Futaleufú River, 43°S) showed affinity to more lineages (Di Prinzio et al. 2015) than a population on the Atlantic slope (Santa Cruz River, 50°S; Ciancio et al. 2015), suggesting that escapes from net-pen aquaculture activity in the Lake District region closer to Futaleufú River would have augmented the genetic diversity initially introduced by open-ocean ranching operations (Riva-Rossi et al. 2012; Di Prinzio et al. 2015). Therefore, in contrast to New Zealand, Patagonian Chinook salmon represent a polyphyletic assemblage, offering new and interesting research avenues to study colonization dynamics and contemporary evolution. Although our results are mostly concordant with the few available molecular studies regarding polyphyletic ancestry, we identified a somewhat different array of contributing lineages. We also identified a methodological anomaly not previously recognized in studies of this kind.

We (1) analyzed the genetic diversity and population structure of Chinook salmon in four western Patagonia watersheds; (2) compared these to native North America populations; and (3) investigated the ancestry and lineage distribution of Chinook salmon populations within and outside open-ocean ranching and net-pen aquaculture regions. We used two different classes of genetic mixture analysis for estimating ancestry, conditional maximum likelihood (CML) and model-based clustering (M-BC). We took advantage of a large, interagency, microsatellite baseline as our known-origin reference against which we compared fish from Patagonia. The Genetic Analysis of Pacific Salmonids (GAPS) consortium assembled a comprehensive genetic baseline for coast-wide fishery management applications involving composition of mixed-stock fisheries (Seeb et al. 2007). The accuracy and precision of mixture analysis using the GAPS baseline is substantial with either conditional or unconditional Bayesian methods (Seeb et al. 2007; Anderson et al. 2008; Hess et al. 2014; Moran et al. 2014), and provides a potentially useful approach for inferring the ancestry of Patagonian Chinook salmon. CML mixture analysis was also used in the two recent studies of the ancestry of Patagonian Chinook salmon mentioned above (Ciancio et al. 2015; Di Prinzio et al. 2015). However, as far as we know, the validity of this application in mixed-origin populations has not been demonstrated. Introduced populations violate the fundamental assumption of the model—that individuals in the unknown “mixture” actually originated from one of the baseline populations. Newly founded populations are isolated and expected to diverge from their ancestors through founder effects and genetic drift (Ciancio et al. 2015). An even greater concern is interbreeding of mixed-origin fish in the new range (admixture), producing novel genotypes not accurately attributable to any single source population. Founding effects and drift are not easily accounted for, but neither are they likely to fundamentally confound our analysis. When considering microsatellites or other neutral markers, it is unlikely that founding and drift would result in a Patagonian population looking more like an unrelated population than the true population of origin. Such a result would require parallel patterns of allele

frequency convergence at multiple highly polymorphic loci. The problem of interbreeding is more serious. It was not clear to us how the Rannala and Mountain (1997) CML mixture algorithm would treat individuals of hybrid origin. Simulations and sensitivity analysis helped us answer that question, and M-BC allowed an independent evaluation of CML mixture analysis in this unusual application.

Materials and methods

Field collection

Chinook salmon populations were sampled in the fall of 2004 from rivers in the four Andean watersheds of Toltén, Petrohué, Aysén and Baker, all draining to the South Pacific through Chile (39-45°S; Figure 1). The results presented here are from 83 fish (after exclusion of 13 fish for which DNA could not be extracted or genotyped). Adult spawners made up 91% of our sample (weight range 2250-21000g), and 9% were juvenile parr (9-37g). Collection took place near spawning habitat using a variety of methods including gillnets, hooks, hand-line, and fly-fishing. A fin clip was dissected from each fish and preserved in 96% ethanol for later genetic analyses. A more detailed description of the sampling and sites is available elsewhere (Correa and Gross 2008).

Population genetic analysis

DNA was extracted from individual fish tissue samples and genotyped for 13 highly polymorphic microsatellite loci. We used the GAPS Chinook salmon microsatellite markers following the GAPS consortium conventions for standardization of genotypic data (Seeb et al. 2007). We calculated allelic and genotypic frequencies for our samples and compared diversity and F statistics with North American populations. Before proceeding with our population genetic analyses, we tested for loci that departed from neutral expectation in order to avoid loci that might bias our parameter

estimates and genetic distance estimates (although departures from neutrality would not necesarily bias our genetic mixture analysis). We used the F_{ST} outlier approach (Beaumont and Nichols 1996) implemented in the LOSITAN software package (Antao et al. 2008). Genetic differences among the South American river basins were analyzed in more detail utilizing pair wise F_{ST} values and leave-one-out jackknife self assignment among introduced populations in order to characterize genetic connectivity and levels of diversity among the introduced Patagonian populations. Because our sample sizes were small, we were especially cautious of non-significant results because we had little power to reject null hypotheses, even when false. However, where we did observe statistical significance, we generally trusted our results as biologically meaningful, despite small sample sizes.

North American baseline dataset

We relied on a baseline dataset of North American reference populations that were selected from the GAPS baseline (Seeb et al. 2007) to represent as closely as possible historical phylogeographic lineages in North American Chinook salmon. Specifically, we used a slight modification of the dataset analyzed by Moran et al. (2013). We added Cowlitz River spring and fall-run populations, and the Green River fall-run population from the West Cascade spring and fall-run reporting groups, and University of Washington Hatchery fall-run population from the South Puget Sound fall-run reporting group, because these specific populations were potential sources of Patagonian Chinook salmon (Table 1). Reporting groups are intended to reflect phylogenetic lineages, and here we use either terminology depending on whether the emphasis is on methodology (reporting groups) or phylogeography (lineages). We excluded the Skykomish River population (South Puget Sound fall reporting group), and the South Umpqua Hatchery population (Mid Oregon Coast reporting group) from the baseline dataset, because these highly diverse populations were genetically intermediate to multiple reporting groups and might be prone to receive spurious individual assignments or fractional

allocation of admixed individuals (mixed ancestry related to multiple founding populations, see below *Simulated mixed-origin founding and mixture analysis*). [Similarly, based on 96 SNPs, Clemento et al. (2014) found the lowest rate of correct assignments in the Mid Oregon Coast reporting group.] There were no historical records of transfers from these populations, and in each case, we retained other, genetically similar populations to represent those North American lineages. Overall, our baseline dataset comprised 19,973 individuals from 146 populations nested in 46 reporting groups distributed from Central Valley, California (40°N) to Southeast Alaska, to the Yukon River in British Columbia (64°N) (Figure 1).

Conditional maximum likelihood mixture analysis

Two principal methods were used to explore the likely ancestry of current introduced Chinook salmon populations in Chile, conditional maximum likelihood (CML) and model-based clustering (M-BC). As a first approximation of genetic similarity and potential ancestry, we used the Rannala and Mountain (1997) algorithm implemented in the ONCOR software package (Kalinowski et al. 2007). That analysis allowed us to estimate the posterior probability distribution for population and reporting group membership (genetic lineage) of each individual fish collected in Patagonia relative to the baseline reference dataset. Individual fish were assigned to the lineage for which they had the maximum *a posteriori* probability of membership. The mean probability across individuals for membership to a particular population was then taken as an approximation of overall genetic ancestral contribution to the population (normally interpreted as the unbiased estimate of that population's contribution to, e.g., a mixed-stock fishery). Simulation studies helped us evaluate the strength of those assumptions. The CML analysis was used as a first approximation to help narrow the range of contributing lineages to the major contributors. A reduced set of populations and lineages was then further analyzed by M-BC.

Simulated mixed-origin founding

Despite the use of CML for studies of ancestry of non-native populations (e.g., Ciancio et al. 2015; Di Prinzio et al. 2015), its validity in that application has not to our knowledge been demonstrated. As a test of the algorithm's sensitivity to the presence of mixed-origin founding, we simulated a new population derived from large and equal numbers of individuals from two of our North American baseline populations that appeared associated with the introduction to Patagonia (Cowlitz River Hatchery spring-run population in the West Cascade spring-run lineage and Soos Creek Hatchery fall-run population in the South Puget Sound fall-run lineage). We then drew 1000 individuals from that new hybrid population at equilibrium, and estimated the posterior probability distributions for those simulated individuals, the same as we did for the Patagonian fish. We sought to determine whether the hybrid-origin genotypes would assign with high probability to one or the other source population, or split their probability of assignment between the two source populations. These novel genotypes might even assign to other, unrelated populations in the large coast-wide North American baseline (where "assignment" simply reflects the maximum *a posteriori* probability of membership to population or reporting group).

Model-based clustering

Based on the results of our CML mixture analysis we conducted a more focused model-based cluster analysis (M-BC) of the introduced fish relative to the putative source populations (computer program Structure; Pritchard et al. 2000). From the North American reference baseline described above, we selected 8,228 fish from 31 populations from seven lineages to include in our analysis, as well as the 83 fish from the four Patagonian populations. Selected North American lineages for M-BC included the following: Whidbey Basin, S. Puget Sound fall, Interior Columbia Basin

summer/fall, Willamette River spring, West Cascade fall, West Cascade spring, and North Oregon Coast. The North American populations and lineages selected for M-BC were those identified as major contributors by virtue of *c.* 10% allocation or higher to any single Patagonian population (North Oregon Coast was included because allocation to that lineage was quite close to our threshold, arguably rounding up to 10%. Including it seemed to be conservative, especially with records of potential introductions of Northern Oregon). This value was selected based on apparent break in the composition estimates. We also inferred from simulations that allocations of less than 10% might be due at least in part to spurious assignment of mixed-origin fish (see *Simulated mixed-origin founding and mixture analysis*).

For the M-BC analysis, we conducted 110,000 MCMC realizations per chain, discarding the first 10,000 iterations as a burn in. Apparent convergence of diagnostic parameters was observed within the first 10,000 iterations (i.e., α, F, the divergence distances among populations $D_{i,j}$, and the likelihood estimate). We used an admixture model, including location information (Hubisz et al. 2009), with allele frequencies correlated among populations (Falush et al. 2003). We assumed population specific F_{ST} values, and updated allele frequencies by using baseline individuals only (thus treating Patagonian samples as having unknown origins). Multiple MCMC chains were constructed for each value of K (number of ancestral clusters).

Heuristic examination of allele frequencies

Finally, we evaluated whether our modeling results made sense heuristically. Allele frequency distributions in putative source populations were examined relative to the introduced populations. We also looked for cases that might disqualify a source, e.g., an allele at high frequency in Patagonia that was not observed in large samples of the putative source populations. Again, this was

not a statistical test, but rather a simple examination of intuitive expectation. Given the complexity of genetic mixture modelling, such an approach seemed warranted.

Results

Data quality, neutrality, and genetic structure

Data quality and genotyping success were high, with an average of 12 out of 13 loci scored per individual fish. We removed from the analysis 4 Aysén individuals that gave 3 or fewer scorable loci, and 9 Toltén individuals that gave no reliable genotypes (collected as decomposing carcass samples), leaving 83 individual Patagonian Chinook salmon for our study.

*Ots*213 departed significantly from neutral expectation (F_{ST} outlier test) and was removed from further population genetic analyses due to potential directional selection (but was retained for genetic mixture analysis, which is generally robust to departures from neutrality). *Oki*100 and *Ots*208b also departed from neutral expectation but were only marginally significant and so were retained. None of these loci are known to diverge from neutral expectation in North American populations (Moran et al. 2013).

Pairwise allele frequency differences were not significant between sample collection locations within river basins in Patagonia and were therefore pooled for population-level analyses (but see below). The analyses we present here are based on those river-basin-level aggregates of collections that are intended to represent separate populations (though because our samples are small, we test and evaluate that assumption several ways). For example, mean F_{IS} values for Patagonian populations were substantially larger than similar estimates from North America (Moran et al. 2013). Those heterozygote deficits might indicate a Wahlund effect of having sampled distinct populations within a river basin. Although departures from Hardy-Weinberg expectations were non-significant, we recognize there was

little power with such small sample size. Non-significant heterozygote deficits were observed at *Ots*201 and *Ots*213 (again, *Ots*213 was not included in most population genetic analyses because of its highly significant departure from neutral expectation).

Genetic diversity

Average genetic diversity in the Patagonian populations (mean H_S = 0.836, SD = 0.128) was nearly identical to contemporary North American populations (mean HS = 0.840, SD = 0.037; Moran et al. 2013). Similarly, average allelic richness in Patagonian populations (10.8, SD = 3.88) was only slightly lower and not significantly different from that observed in a coast-wide North American sample (13.3, SD = 1.81; Moran et al. 2013). Allelic richness was nearly identical to the North American populations that were identified as likely contributors (see below, *e*.g., Whidbey Basin, West Cascades, and Oregon Coast, 10.3, SD = 4.19). The alleles observed in Patagonia included more than half of those ever recorded in North America, from the Yukon River in the Bering Sea to Central Valley California (most of the native species range).

Among populations in Patagonia, overall genetic diversity did not differ significantly, as inferred from heterozygosity and allelic richness. Even though our population samples from Patagonia were quite small, we seem to have been successful in capturing a broad cross section of genetic diversity in these introduced populations.

Population structure

Between Patagonian river basins, allele frequencies were highly significantly different (mean pair-wise P = 0.0009, where Bonferroni-corrected α = 0.00833). Similarly, pairwise F_{ST} values were large among Patagonian populations (mean F_{ST} = 0.039, SD = 0.004), especially considering the

narrow range of genetic diversity among the putative source populations (F_{ST} = 0.028, SD = 0.006) relative to all of North America (F_{ST} = 0.063, SD = 0.01).

Contingency testing, pair-wise F_{ST} estimates, and jack knife self-assignment analysis were all consistent in showing the closest genetic relationship between the two southern populations Aysén and Baker (F_{ST} = 0.01). Petrohué was similar to Baker and Aysén, whereas Toltén, the northern-most population, was the most distinct of the four (F_{ST} = 0.065).

Stock composition of Patagonian Chinook salmon (CML mixture analysis)

We identified multiple North American lineages in Patagonia. Of the 46 reporting groups, 16 (35%) were identified by CML mixture analysis as possibly represented in Patagonia (Fig. 1, Table 2). However, based on our simulations, we expected a small fraction of spurious assignments/allocation associated with admixture among lineages occurring in the introduced range (see below, *Simulated mixed-origin founding and mixture analysis*). Hence, in order to emphasize mayor contributing lineages and avoid over-interpretation, we focused on putative contributors with at least *c.* 10% allocation from any single study site (Table 2; Figure S2(B)). Seven reporting groups satisfied this criterion. Approximately south to north in their native range, these were: Willamette River spring, North Oregon Coast, West Cascade fall, West Cascade spring, Interior Columbia Basin summer/fall, South Puget Sound fall, and Whidbey Basin.

The estimated number of lineages that contributed to our South American populations was higher in the north (Toltén and Petrohué) than in the south of our study area (Aysén and Baker) (Figure 1). West Cascade fall, and especially West Cascade spring, contributed substantially, especially in the south with 71% and 65% genetic contribution of West Cascade spring to Aysén and Baker populations (Table 2). Interior Columbia basin summer/fall Chinook salmon contributed to all four Patagonian samples, but especially to those from the north. Other contributors showed more localized effects. For

example, South Puget Sound fall in Petrohué (20%), Whidbey Basin in Toltén (20%), and Willamette River spring in Baker (18%). Various other donors had lower contributions, but, again, some of those results might be attributable to the misassignments we observed in the simulation of mixed ancestry.

Whereas the two northern river basins (39°-42°S) appeared highly polyphyletic, the two southern basins (45°-46°S) were nearly monophyletic, attributing nearly all of their ancestry to the closely related Lower Columbia River West Cascade and Willamette River lineages. We found a general congruence between the stock composition estimates and the proportions of individual fish assignments (Table 2; Supporting Information, Table S2).

Split assignments consistent with primary donors

The ancestries of most fish were readily identifiable though CML mixture analysis. Most assigned with high probability to one of the baseline reporting groups (average maximum *a posteriori* value = 0.903, SD = 0.145) or reference populations (0.861, SD = 0.168) (Figure 2a; for population-level assignment probabilities, see Supporting Information, Figure S1). High assignment probabilities to baseline reporting groups were observed even in watersheds where spawners from multiple lineages were collected together, and interbreeding was apparent (*i.e.*, Toltén and Petrouhé). Both at the reporting group and population-level, individual fish that did show affinity to multiple source lineages, were invariably associated with the same sources to which other fish in the same collection assigned with high probability. For example, Petrohué and Toltén had individuals that assigned with relatively high probability to North Oregon Coast, West Cascade spring and fall, Interior Columbia Basin summer/fall, and South Puget Sound fall; however other presumptive hybrid individuals split their assignment probability among those same sources.

Simulated mixed-origin founding and mixture analysis

Our simulation result showed that most individual fish in a population derived from multiple sources would assign back to those sources, often splitting their assignment probability between source populations (Figure S2). However, we also learned that sometimes a non-trivial number of simulated, mixed-origin fish might assign with high probability to unrelated populations and lineages. Some misassignment was expected to genetically similar reporting groups. Both reporting groups used in our simulation have closely related sister groups in the GAPS baseline. West Cascade spring is genetically similar to West Cascade fall, and South Puget Sound fall is similar to Whidbey Basin. Most simulated fish (61.3%) assigned to one or the other of the true source reporting groups. Another 28.2% assigned to those closely related sister groups. That level of misassignment was expected based on leave-one-out jackknife analysis of the North American baseline. Approximately 19% of real fish collected from Cowlitz River spring (one of the seed populations for the simulation) misassigned to Cowlitz River fall in the West Cascade fall reporting group (REF).

Expected misassignment to closely related reporting groups contrasted strongly with assignment of simulated individuals to unrelated groups. In our simulation, 10% of individuals assigned to unrelated reporting groups, 44% of these with high assignment probability ($P \geq 0.8$). Interior Columbia Basin summer/fall received about half of these misassignments, whereas the rest distributed in fourteen other unrelated reporting groups.

Our simulation study confirmed the general utility of CML mixture analysis for individual assignment and proportional allocation despite admixture, but also showed the potential for misleading results, even when individuals assign with high probability. Therefore, we interpreted our empirical results of CML mixture analysis of real fish with caution when the estimated proportional contribution of a reporting group approached the level of misassignment we observed in the simulation (i.e., <10%, see above *Stock composition of Patagonian Chinook salmon*). Any criterion would be somewhat

arbitrary, but for the empirical dataset there seemed to be a break between 0.08 and 0.1, and we knew that values below 0.08 could be relatively strongly influenced by spurious assignments.

A heuristic examination of the estimated posterior probability density for real versus simulated individuals suggested there might be fewer intermediate assignments in the Patagonia fish (split probabilities between reporting groups) than expected for mixed-origin populations at equilibrium (e.g., the simulated mixed-origin population). If so, it might be the result of assortative mating or other non-equilibrium conditions. As indicated above, most of the fish from Patagonia (81.9%) assigned to putative North American baseline reporting groups with greater probability than 0.8, and only 5 fish (6%) had assignment probabilities less than 0.6 (Figure 2). Therefore, the point estimate for mean assignment probability was higher for Patagonian fish than expected for mixed-origin populations at equilibrium, and the distribution was narrower but not significantly so (overlapping 95% confidence intervals). Although inconclusive regarding equilibrium and random mating, the simulations confirmed the utility of the CML algorithm for studies of ancestry with the important caveat that a few individuals can show high relative probability of membership to completely unrelated populations in distinct lineages.

Equivocal results regarding expected contributors

Not a single fish we sampled from four locations in Patagonia showed any affinity whatever to the University of Washington Hatchery fall-run stock (zero relative probability of membership). Only five fish, collected in the Petrohué basin, assigned with high probability to another Puget Sound fall-run hatchery stock, Soos Creek (from which University of Washington broodstock was derived), but none were similar to our contemporary sample from University of Washington Hatchery fall-run stock.

Central Valley California was also a suspected source of Patagonian Chinook salmon, however, our results offered very little support for that conclusion at the locations we sampled. Only two fish assigned to Central Valley populations, one to Tuolumne River and the other to Stanislaus River. It was unclear if those two fish demonstrated true Central Valley ancestry or spurious assignments resulting from admixture (see above *Simulated mixed-origin founding and mixture analysis*). An apparent lack of Central Valley ancestry was similar to the findings of one previous molecular study (Ciancio et al. 2015) but different to another (Di Prinzio et al. 2015). It is worth noting that previous studies like ours did not account for potential spurious assignments of mixed-origin fish to unrelated reporting groups. Thus, numerous additional sources were identified as putative contributing lineages. We do not refute the presence of these additional lineages. We simply recognize the limitation we discovered in our genetic mixture analysis regarding mixed-origin fish, and we focus on what we take to be the major lineages that became established at our study sites (those lineages receiving ≥ 10% allocation).

Model-based clustering (M-BC)

Our reduced reference dataset for M-BC included 31 of the GAPS baseline populations (Moran et al. 2013) from seven reporting groups (Whidbey Basin, S. Puget Sound fall, Interior Columbia Basin summer/fall, West Cascade fall, West Cascade spring, North Oregon Coast, and Willamette River spring). These reporting groups contributed most (84.13%) of the combined posterior probability in the reporting group-level CML mixture analysis (Table 2). Examination of log likelihood of the modeled clusters (Pritchard et al. 2000) for various numbers of clusters (K) in the North American populations showed a peak in delta log likelihood at $K = 3$ and $K = 5$ (Evanno et al. 2005; Earl and vonHoldt 2011). We observed the clearest discrimination of the seven reporting groups at $K = 5$, and that value showed high concordance with the genetic stock reporting groups that we used in the

CML mixture analysis (Figure 3a). Concordance was also seen between the mean *Q*-value proportions in the Patagonian populations and the genetic stock mixture proportion estimates based on posterior probability density from CML mixture analysis (compare Table 2 and Figure 3). For example, from south to north, individual Baker fish showed *Q*-value proportions mostly consistent with ancestry from West Cascade spring/fall (Q3), or a combination of West Cascade spring and Willamette River spring runs (Q4). Aysén fish mostly showed affinity with West Cascade spring/fall runs (Q3), but also some possible Willamette River spring (Q4) and little South Puget Sound fall and Whidbey Basin influence (Q5). In Petrohué, West Cascade spring/fall and Willamette River (Q3 and Q4) lineages were still important, but a substantial increase of all other lineages was apparent, particularly South Puget Sound fall and Whidbey Basin (Q5). This trend towards more diverse ancestry in the north of the Patagonian range persisted in Toltén where fish showed affinity to all five ancestral lineages inferred by the M-BC analysis (Q1 to Q5), and by extension the seven lineages distinguished in phylogeographic analysis of Chinook salmon in the native range (Moran et al. 2013). Thus, we observed a spatial gradient in the ancestry of Patagonian Chinook salmon populations (Figure 3b). Model-based clustering appeared more sensitive to the discrimination of West Cascade spring versus fall run but less able to differentiate Willamette River ancestry from West Cascade spring run. In general, model based clustering suggested broader, more uniform contributions from the same source populations that were identified with CML mixture analysis. Beyond that, however, results from M-BC were highly concordant with CML and those results led to nearly identical inferences about the source populations that were introduced into Patagonia.

Heuristic examination of allele frequencies

For every locus, allele frequencies for nearly all 259 alleles in the Patagonian populations were consistent with the distributions observed in the putative populations of origin. The most common alleles in the Patagonian populations were also common in the North American source populations identified here. All alleles that were present in the Patagonian populations were also observed in the top putative North American source lineages.

Discussion

We investigated the phylogenetic ancestry of introduced Chinook salmon in Patagonia by conducting two different classes of genetic stock identification methods. Diverse genotypes led to the identification of many putative ancestral sources introduced primarily from the states of Oregon and Washington. Our results were largely consistent with historical records of fish introductions and recent molecular genetic studies. However, we also found interesting differences such as apparent contributions from undocumented introductions, and conversely, lack of evidence supporting the naturalization of well-documented introductions.

Our study is distinguished from previous molecular studies of Patagonian Chinook salmon (Becker et al. 2007; Riva-Rossi et al. 2012; Ciancio et al. 2015; Di Prinzio et al. 2015) in three principal ways: 1) We used the most inclusive baseline dataset possible, including all potential North American donor lineages. Previous studies have been more or less limited by the number of reference lineages available for particular genetic markers. The GAPS microsatellite baseline is the most extensive of its kind and is ideally suited for this application. 2) To our knowledge, ours is the first study of its kind to take into account the potential for spurious allocation of admixed genotypes. Other studies have addressed genetic drift in introduced populations (Ciancio et al. 2015), but not hybridization between divergent lineages. We suggest that admixture may be more problematic than genetic drift, and simulations of mixed-origin founding offered a cautionary note on potentially

spurious results. Our observations are relevant to many studies that seek to characterize small genetic contributions from multiple founding lineages. 3) Our study included naturalized populations both within and outside salmon aquaculture zones. This helped clarify the poorly documented role of net-pen aquaculture on Chinook salmon introduction to Chilean Patagonia. Thus, our study provides important new insight into the ancestry and patterns of introduction of Patagonian Chinook salmon.

Non-equilibrium conditions and potential assortative mating

Allele frequency differences between sample collections within Patagonian basins were non-significant, and samples were pooled into putative populations. Those basin-level pooled samples were highly significantly different in allele frequency and showed high levels of individual assignment to collection location. Because our samples were small it was important to evaluate the strength of assuming basin-level population structure from several different perspectives. In addition to testing for allele-frequency differences between collections and between basins, we also tested for departures from expected Hardy-Weinberg genotypic proportions, especially heterozygote deficits that might indicate a Wahlund Effect resulting from sampling two or more genetically distinct populations. This analysis would help us evaluate population structure and potential assortative mating among sympatric founding lineages. We did find elevated F_{IS} values within basins (putative populations), but the F_{IS} estimates were not significantly greater than zero. Recognizing limited power for detecting a Wahlund Effect, we also tried to draw inference from CML individual assignment probabilities. Fewer intermediate assignments (split probabilities between reference groups) were observed than expected for a mixed-origin population at equilibrium. If real, such a result might be consistent with, for example, assortative mating of two or more genetically distinct lineages. Alternatively, strong out-breeding depression (e.g., Gharrett and Smoker 1991) or insufficient time since introductions could result in fewer than expected hybrids. The point estimate for mean assignment probability was higher for Patagonian fish and the

distribution was narrower but again not significantly so. We conclude that evidence for non-equilibrium conditions is ambiguous (*e.g.*, assortative mating, on going range expansion, etc.). Resolution of this question will simply require larger sample sizes within and among sites and river basins as well as across multiple brood years and across generations. Alternatively or additionally, more markers could be surveyed. For example, genome sequencing would provide haplotype arrays that might be quite powerful for evaluating introgression and equilibrium.

Diversity of founding lineages uncorrelated with population genetic diversity

Population genetic diversity measured by heterozygosity and allelic richness was high in Patagonia—nearly identical to North American populations. Our genetic CML mixture analyses also suggested a diverse array of founding lineages in Patagonia. Initially, we assumed high genetic diversity, i.e., heterozygosity and allelic richness, was a result of mixed ancestry. Other studies have made similar conclusions (Riva-Rossi et al. 2012; Ciancio et al. 2015; Di Prinzio et al. 2015). CML mixture analysis and M-BC both estimated increasing diversity of founding lineages from south to north in Patagonia. Baker and Aysén, in the south, had low lineage diversity, whereas Petrohué and Toltén, in the north, had a high diversity of founding lineages. Surprisingly, however, populations with the most diverse ancestry showed no higher levels of heterozygosity or allelic richness. Nor was there a spatial gradient for heterozygosity or allelic diversity, as was evident for lineage diversity. This also suggests fewer-than-expected hybrids (see *Non-equilibrium conditions and potential assortative mating*).

It would seem almost axiomatic that a diversity of founders would introduce more alleles relative to monophyletic populations, yet other factors might be confounding a clear association between lineage diversity and genetic diversity. Given our broad and overlapping estimates of

heterozygosity and allelic richness, it is likely that our sample sizes were too small to obtain an accurate or precise view of genetic diversity relative to lineage diversity.

Spurious individual assignments related to mixed-origin populations

A principal challenge in this study was to distinguish the genetic signal of true ancestry from the noise created by mixed origin, introgressed populations. In our CML mixture analysis we expected some misassignments between related populations. The patterns of misassignment in the North American reference baseline were well understood based on previous results (Hess et al. 2014). However, our simulation of mixed-origin founding revealed some spurious genetic allocation not previously reported in studies that used this approach (Ciancio et al. 2015; Di Prinzio et al. 2015). In the empirical results, we observed allocations of up to 6% estimated genetic contribution from very unlikely donor regions, such as Nass River and the South Thompson River, both in British Columbia. This 6% level of contribution is equivalent to a little more than one fish with a high assignment probability in a population sample of the size we collected. The CML simulation showed us that mixed origin individuals (hybrids) usually assign to the true donors, however, some individuals will assign to unrelated populations, sometimes with high assignment probability (Figure S2). Note, however, that the probability values are conditional on available baseline references. A high assignment probability might simply mean that no other reference population could likely have produced a given genotype. This is relevant, for example, to studies that assume a probability threshold (e.g. 0.8) for accurate assignment, and especially to those with incomplete baseline datasets. While a probability threshold such as 0.8 is clearly useful in conventional genetic mixture applications (Moran et al. 2014), in a mixed-origin founding application, it is almost certainly the case that some high-probability individual assignments are attributable to the misassignment phenomenon that we demonstrated through simulation. To avoid falsely implicating potential donor lineages we set a lower threshold for genetic contribution below

which putative ancestry was viewed with caution and some skepticism. Based on review of both simulated and empirical data, we used a threshold of 10% estimated genetic contribution to any single population in Patagonia. It was not that we rejected as potential contributors lineages below 10%, rather we were less confident in identifying those as founders.

Reconciling historical introductions with genetic mixture results

For the most part, our findings confirm the importance of entrepreneurial attempts to establish Chinook salmon open-ocean ranching programs in Chile during the late 1970s and 1980s. In particular, the West Cascade spring and fall lineages, introduced from the Lower Columbia River, were likely associated with the enterprises suspected to have initiated the invasion. In 1978 Domsea Pesquera Chile Ltda. (a subsidiary of Union Carbide Corporation, USA) began yearly stockings of Chinook salmon in a small stream on Quinchao Island, Curaco de Vélez, Chiloé (42°S). Two strains of Chinook salmon were introduced (Lindbergh 1982; Anonymous 1989). The primary stock that appeared to become established was Cowlitz River spring run (Lindbergh et al. 1981a,b; Lindbergh 1982); precisely the same primary ancestral lineage we identified in our samples. The second stock used in 1978 was not identified in historical records (but see below).

The finding of the related fall-run lineage (West Cascade fall) in our samples might correspond to the second strain mentioned by Jon Lindbergh, especially because at the time both spring and fall runs were propagated at the Cowlitz River Hatchery. Alternatively, that second stock might have been the Interior Columbia Basin summer/fall lineage propagated at Little White Salmon National Fish Hatchery. Regarding the introductions in Río Chirri (Río Bueno) in the early 1970s from Green River Hatchery (also located on the Cowlitz River), establishment seems unlikely. Although this population is from the West Cascade fall lineage, to our knowledge there are no records of Chinook salmon in Río Bueno during the decade that followed introduction (Correa and Gross 2008), and no

fish in our analyses assigned to Green River Hatchery. Definite confirmation would require additional analyses of samples from Río Bueno.

Subsequent open-ocean ranching experiments in the Magellan region (51°S) would have introduced the University of Washington Hatchery fall-run stock (Donaldson and Joyner 1983). By 1982, the aforementioned enterprise, that had been sold to Fundación Chile and renamed to Salmones Antártica S.A., expanded its operations further south where additional stockings took place, first in Río Santa María (1982) and then in Río Prat (1983-1989). The stocks of choice were University of Washington Hatchery fall-run and the newly established Chilean-based brood stocks (see references in Table 1). Nevertheless, we found little evidence supporting the successful colonization by University of Washington Hatchery fall-run stock introduced in the Magellan region of Chile. This stock, also known as Portage Bay fall-run Chinook salmon, from the South Puget Sound fall reporting group, was primarily derived from the Green River draining to Puget Sound (different to Green River in the West Cascades), particularly from Soos Creek Hatchery (1949-1950s), though exchanges with other populations took place over the years (Quinn et al. 2002). The Soos Creek Hatchery itself had exchanges with many other populations, mainly within the Puget Sound area. It is puzzling that in our study not a single fish showed any probability of assignment to the University of Washington reference population. Only five fish (17% genetic contribution) from Petrohué River assigned to Soos Creek Hatchery.

Previous studies have inferred ancestral affiliation from the University of Washington Hatchery based on mitochondrial DNA (D-loop segments; Becker et al. 2007; Riva-Rossi et al. 2012), but owing to the low resolution of these analyses (incomplete baselines; geographically wide-spread haplotypes), it is unclear if these were real signals. Others, using SNPs and a more extensive baseline (Clemento et al. 2014), found little evidence in support of South Puget Sound ancestry, although they still suggested a possible contribution of University of Washington Hatchery stock based on its

supposed origin in lower Columbia River lineages, which do appear as a major contributors to Patagonian populations (Ciancio et al. 2015; Di Prinzio et al. 2015). Our results provided no support whatever for that interpretation. The GAPS baseline shows no similarity between Lower Columbia River populations and UW—or indeed any other Puget Sound populations. University of Washington Hatchery brood stock is genetically distinct with 80% correct assignment of known-origin fish in leave-one-out jackknife analysis (not shown). All UW fish assign correctly to the South Puget Sound fall run lineage (i.e., no fish misassigned to West Cascade spring or any other population outside South Puget Sound).

Given the historical record and our genetic mixture results, we suggest three possible outcomes for the introductions from University of Washington Hatchery fall-run stock into the Magellan region: (i) Naturalization failed, leaving no genetic traces in current Patagonian populations. (ii) Naturalization succeeded to some extent, and assignments to South Puget Sound fall represent remnant (though admixed) evidence. (iii) Naturalization succeeded in the Magellan region, yet subsequent range expansion did not reach our study area, or those of other studies in Argentinean and Chilean Patagonia (Ciancio et al. 2015; Di Prinzio et al. 2015). In any case, there is little doubt that open-ocean ranching experimental operations in Chile in the late 1970s and 1980s were responsible for the introduction of West Cascade spring Chinook salmon in Patagonia (see also Ciancio et al. 2015; Di Prinzio et al. 2015).

Patagonian Chinook salmon unrelated to the West Cascade spring/fall lineage (hereafter non-WC) accounted for almost half of the fish (45.8%), mostly taken from north of parallel 45°S. The phylogenetic origins of non-WC fish were tracked to 14 diverse and geographically widespread lineages in North America (Figure 1). This seemingly hyperdiverse assemblage is at least partly explained by spurious individual assignments related to mixed ancestry (consistent with our simulations). However, some of this diversity appears due to accidental escapes from thriving Chilean

net-pen salmonid aquaculture operations in the 1990s that imported ova from diverse sources (Table 1). Others also have come to similar conclusions by reference to molecular data (Consuegra et al. 2011; Riva-Rossi et al. 2012; Di Prinzio et al. 2015).

Since 1988, Chilean salmonid aquaculture shifted entirely to net-pen aquaculture. However, substantial escapes were frequent, yet hard to quantify because they commonly went unreported (Soto et al. 2001; Naylor et al. 2005; Buschmann et al. 2006; Arismendi et al. 2009). Data gathered from insurance companies alone (Sepúlveda et al. 2013) lead to an estimated average of *c.* 1 million salmon and trout escaping every year, primarily from net-pen aquaculture. Although Chinook salmon farming has always been marginal in Chile relative to other species (<4,000 tons y-1; <2.5% y-1 of total salmonid production; FAO 2014), sustained Chinook salmon farming in leaky rearing facilities certainly contributed propagules to the wild (Soto et al. 2001). We estimated that for every million escaped salmon per year, adjusting for the contribution of Chinook salmon to annual harvest (FAO 2014), *c.* 100,000 farmed sub-adult Chinook salmon could have escaped between the onset of modern salmonid farming (1988) and the date of our sampling (early 2004). This highly conservative figure illustrates the potential of net-pen aquaculture as a relatively diffuse source of colonists.

During this period, numerous national and trans-national aquaculture companies were established in Chile, propagating Chinook salmon brood stocks to supply increasing demand. Aedo (2011) assembled the fragmentary record of ova importations and concluded that ova originated at least from Washington, Oregon, Vancouver Island, and New Zealand, but specific stocks mostly went undocumented (Table 1). Although sparse, this information could explain the incidence of Patagonian genotypes related to multiple lineages of the West Coast of U.S.A. Furthermore, the spatial distribution of non-WC fish was concentrated in the north (Toltén and Petrohué) and could be the result of more intensive propagule pressure of distinct lineages farmed in this region. In fact, during the 1990s, most aquaculture activity (and escapes) were concentrated in the Lakes District region (41-43°S), within 200

km from Petrohué River mouth. Toltén River (39°S), being a few hundred kilometers further north, could have received colonists dispersing naturally from the Lake District region; or escapees from inland hatcheries; or deliberate stockings, since this is a well-populated region easily accessible by paved roads. In sharp contrast, in the more remote south of our study area (45-50°S), aquaculture activity was only incipient (Aysén River) or non-existent (Baker River). Here, accidental escapes or unreported deliberate stockings were less likely, and natural dispersion alone seems the most likely source of founding Chinook salmon, especially in the remote and isolated Baker River basin.

Emerging patterns of lineage distribution

Northern study sites (Toltén and Petrohué) showed unexpectedly high lineage diversity, including substantial estimated contributions from five or more lineages (North Oregon Coast, West Cascade spring, South Puget Sound fall, Whidbey Basin, and Interior Columbia River summer/fall). By contrast, southern study sites (Aysén and Baker) revealed a striking dominance of three closely related lineages from the Lower Columbia River (i.e., West Cascade spring, West Cascade fall and Willamette spring lineages). A similarly diverse assemblage was also found in the Santa Cruz River draining to the Atlantic Ocean in Argentinean Patagonia (Ciancio et al. 2015). A number of possibilities could explain this interesting pattern: (1) As discussed above, higher propagule pressure of distinct lineages leaking from fish farms might have resulted in higher lineage diversity in northern sites compared to remote southern localities (propagule pressure hypothesis). (2) Most dispersing colonists moved northwards in our study area such that those colonizing Aysén and Baker rivers were derived from stockings in the Magellan region of West Cascade spring/fall ancestry, whereas those colonizing Petrohué and Toltén rivers were derived from fish farms in the Lake District region (propagule+dispersion hypothesis). This pattern would resemble natural ocean migration patterns of Washington and Oregon stocks towards the north in the North Pacific, although it is unclear how the fish would respond to magnetic stimuli of the

southern hemisphere (Putman et al. 2014). (3) Deliberate stockings initiated the first wave of invasion that eventually reached Aysén and Baker rivers, either from the north or south. Previous establishment of West Cascade spring/fall lineages might have inhibited the expansion of farmed lineages from the north (density-dependent hypothesis) (Urban and De Meester 2009; Waters et al. 2013, e.g., Eschbach et al. 2014). At present, it is hard to gauge the relative importance of these processes, and it is conceivable that each contributed to the observed pattern of lineage distribution. Future work should increase the geographical coverage, number of samples and sample sizes, and incorporate time-series to study the consequences of anthropogenic sequential introductions, dynamics of range expansion, and lineage mixing and admixture.

Concluding remarks

Our study provides the most comprehensive assessment to date of ancestry in Patagonian Chinook salmon. The results indicated that multiple deliberate and accidental introductions related to discontinued open-ocean salmon ranching and net-pen aquaculture in Chile (1970s-2000s) contributed to the establishment of a diverse Chinook salmon assemblage. Furthermore, substantial population structure and distinct lineage patches were observed between the northern (39-42°S) and southern (45-46°S) range, which were areas differentially affected by aquaculture activity.

Complex invasion dynamics and distinct patterns of genetic diversity are likely to have profound evolutionary consequences in the continuing range expansion of Chinook salmon in Patagonia. High genetic diversity, as measured by high allelic and lineage richness (this study; Di Prinzio), high phenotypic plasticity and rapid adaptive evolution observed in Chinook salmon introduced to New Zealand (Quinn et al. 2001; Kinnison et al. 2008), as well as apparent pre-adaptation to environmental conditions encountered in Patagonia (Correa and Gross 2008), are all features that suggest very high adaptive potential of Chinook salmon in Patagonia. Lower-than-expected levels of

heterozygosity, if confirmed and maintained over generations, could indicate reproductive isolation between sympatric lineages, which would help maintain lineage identity. Whether this happens is crucial to the evolutionary future of the species in its new range. Lineages could either evolve adapting genetically to local conditions (e.g., Narum et al., submitted to Evolutionary Applications; see also the case of New Zealand, Kinnison et al. 2008) or coalesce forming completely new varieties with unexpected eco-evolutionary dynamics. Chinook salmon may exert strong ecological impacts in freshwater, estuarine and marine ecosystems (Correa and Gross 2008), and different evolutionary outcomes will likely mediate the kind and intensity of these impacts owing to considerable phenotypic variation among lineages (Kinnison et al. 2008).

The involvement of the still-expanding Chilean commercial net-pen salmonid aquaculture industry on Chinook salmon naturalization seems now irrefutable (cf. Soto et al. 2001; Correa and Gross 2008). All evidence presented herein is consistent in this regard (see also Di Prinzio et al. 2015). Similarly, Consuegra *et al.* (2011) demonstrated the introgression of cultured rainbow trout genes into pre-existing, naturalized rainbow trout populations in Patagonia. In other continents, aquaculture escapes raise issues mainly regarding genetic and ecological effects on populations of wild conspecifics (Naylor et al. 2005; Jensen et al. 2010). However, in Patagonia issues are fundamentally different and much less understood (Sepúlveda et al. 2013). Escapes resulted in rapid propagation in ecosystems naturally devoid of salmonids, an apex predator with keystone attributes, whose ecologic and socioeconomic impacts merit urgent research. Chinook salmon has disappeared from captive rearing in Chile (FAO 2014), and yet its eco-evolutionary legacy will last indefinitely. Other heavily cultivated species such as coho salmon (*O. kisutch*) and Atlantic salmon (*S. salar*) might follow similar trajectories.

Salmonid escapes from rearing facilities in Chile compromise the sustainability of the salmonid aquaculture industry (Buschmann et al. 2009; Sepúlveda et al. 2013). It is of uttermost importance that the industry assumes responsibility, and improves operational standards. This can only be achieved if policy-makers in Chile improve out-dated regulatory frameworks and strengthen enforcement, in order to implement sound, science-based technical and governance solutions (Buschmann et al. 2009; Jensen et al. 2010; Sepúlveda et al. 2013). Finally, we urge the creation of aquaculture-free zones appropriate for native biodiversity conservation and future baseline reference (Consuegra et al. 2011; Arismendi et al. 2014).

References

Aedo, E. 2011. Información sobre siembras de salmónidos en el ambiente natural e incidencia de escapes desde centros de cultivo en la Región de Aysén, referenciada geográficamente. Pp. 120–135 *in* E. Niklitschek and P. Toledo, eds. Evaluación cuantitativa del estado trofico de salmonidos de vida libre en el fiordo Aysén, XI región. Informe final proyecto FIP200830. Universidad Austral de Chile - Subsecretaría de Pesca, Puerto Montt.

Anderson, E. C., R. S. Waples, and S. T. Kalinowski. 2008. An improved method for predicting the accuracy of genetic stock identification. Can. J. Fish. Aquat. Sci. 65:1475–1486.

Anonymous. 1989. Coho in Chile: An interview with Jon Lindbergh. Egg&Smolt 2.

Antao, T., A. Lopes, R. J. Lopes, A. Beja-Pereira, and G. Luikart. 2008. LOSITAN: A workbench to detect molecular adaptation based on a F_{st}-outlier method. BMC Bioinformatics 9:323.

Arismendi, I., B. E. Penaluna, J. B. Dunham, C. Garcia de Leaniz, D. Soto, I. A. Fleming, D. Gomez-Uchida, G. Gajardo, P. V. Vargas, and J. León-Muñoz. 2014. Differential invasion success of salmonids in southern Chile: patterns and hypotheses. Reviews in Fish Biology and Fisheries 24:919–941.

Arismendi, I., D. Soto, B. Penaluna, C. Jara, C. Leal, and J. León-Muñoz. 2009. Aquaculture, non-native salmonid invasions and associated declines of native fishes in Northern Patagonian lakes. Freshwater Biology 54:1135–1147.

Asif, J. H., and P. J. Krug. 2012. Lineage distribution and barriers to gene flow among populations of the globally invasive marine mussel *Musculista senhousia*. Biol Invasions 14:1431–1444.

Astorga, M. P., C. Valenzuela, I. Arismendi, and J. L. Iriarte. 2008. Naturalized Chinook salmon in the northern Chilean Patagonia: Do they originate from salmon farming? Revista de Biología Marina y Oceanografía 43:669–674.

Basulto, S. 2003. El largo viaje de los salmones. Una crónica olvidada. Propagación y cultivo de especies acuáticas en Chile. Maval Ltda., Santiago.

Beaumont, M. A., and R. A. Nichols. 1996. Evaluating loci for use in the genetic analysis of population structure. Proc. R. Soc. Lond. B 263:1619–1626.

Becker, L. A., M. A. Pascual, and N. G. Basso. 2007. Colonization of the Southern Patagonia ocean by exotic Chinook salmon. Conservation Biology 21:1347–1352.

Buschmann, A. H., F. Cabello, K. A. Young, J. Carvajal, D. A. Varela, and L. Henríquez. 2009. Salmon aquaculture and coastal ecosystem health in Chile: Analysis of regulations, environmental impacts and bioremediation systems. Ocean & Coastal Management 52:243–249.

Buschmann, A. H., V. A. Riquelme, M. C. Hernandez-Gonzalez, D. Varela, J. E. Jimenez, L. A. Henriquez, P. A. Vergara, R. Guinez, and L. Filun. 2006. A review of the impacts of salmonid farming on marine coastal ecosystems in the southeast Pacific. ICES Journal of Marine Science 63:1338–1345.

Ciancio, J. E., C. R. Rossi, M. Pascual, E. Anderson, and J. C. Garza. 2015. The invasion of an Atlantic Ocean river basin in Patagonia by Chinook salmon: new insights from SNPs. Biol Invasions, doi: 10.1007/s10530-015-0928-x.

Clemento, A. J., E. D. Crandall, J. C. Garza, and E. C. Anderson. 2014. Evaluation of a single nucleotide polymorphism baseline for genetic stock identification of Chinook Salmon (*Oncorhynchus tshawytscha*) in the California Current large marine ecosystem. Fishery Bulletin 112:112–130.

Consuegra, S., N. Phillips, G. Gajardo, and C. G. de Leaniz. 2011. Winning the invasion roulette: escapes from fish farms increase admixture and facilitate establishment of non-native rainbow trout. Evolutionary Applications 4:660–671.

Correa, C., and M. R. Gross. 2008. Chinook salmon invade southern South America. Biological Invasions 10:615–639.

Crawford, S., and A. Muir. 2007. Global introductions of salmon and trout in the genus *Oncorhynchus*: 1870–2007. Reviews in Fish Biology and Fisheries 18:313–344.

Deans, N., M. Unwin, and M. Rodway. 2004. Sport fishery management. Pp. 41.1–41.16 *in* J. Harding, P. Mosley, C. Pearson, and B. Sorrell, eds. Freshwaters of New Zealand. New Zealand Hydrological Society and New Zealand Limnological Society, Wellington.

Del Real, A. 1993. Antecedentes sobre el cultivo de salmón Chinook (*Oncorhynchus tshawystcha* W.) en la fase de agua dulce proveniente de reproductores retornantes en la piscicultura experimental Lago Rupanco. Universidad de Los Lagos, Departamento de Acuicultura y Recursos Acuaticos, Osorno, Chile.

Di Prinzio, C. Y., C. R. Rossi, J. Ciancio, J. C. Garza, and R. Casaux. 2015. Disentangling the contributions of ocean ranching and net-pen aquaculture in the successful establishment of Chinook salmon in a Patagonian basin. Environmental Biology of Fishes, doi: 10.1007/s10641-015-0418-0.

Donaldson, L. R., and T. Joyner. 1983. The salmonid fishes as a natural livestock. Scientific American 249:50–58.

Earl, D. A., and B. M. vonHoldt. 2011. STRUCTURE HARVESTER: a website and program for visualizing STRUCTURE output and implementing the Evanno method. Conservation Genet Resour 4:359–361.

Eschbach, E., A. W. Nolte, K. Kohlmann, P. Kersten, J. Kail, and R. Arlinghaus. 2014. Population differentiation of zander (*Sander lucioperca*) across native and newly colonized ranges suggests increasing admixture in the course of an invasion. Evol Appl 7:555–568.

Evanno, G., S. Regnaut, and J. Goudet. 2005. Detecting the number of clusters of individuals using the software structure: a simulation study. Molecular Ecology 14:2611–2620.

Falush, D., M. Stephens, and J. K. Pritchard. 2003. Inference of population structure using multilocus genotype data: linked loci and correlated allele frequencies. Genetics 164:1567–1587.

FAO. 2014. Fishery and aquaculture statistics. Global aquaculture production 1950-2012 (FishstatJ) In: FAO Fisheries and Aquaculture Department [online or CD-ROM]. Rome. Updated 2014. http://www.fao.org/fishery/statistics/software/fishstatj/en.

Gharrett, A. J., and W. W. Smoker. 1991. Two generations of hybrids between even- and odd-year pink salmon (*Oncorhynchus gorbuscha*): a test for outbreeding depression? Can. J. Fish. Aquat. Sci. 48:1744–1749.

Hess, J. E., J. M. Whiteaker, J. K. Fryer, and S. R. Narum. 2014. Monitoring stock-specific abundance, run timing, and straying of Chinook salmon in the Columbia River using genetic stock identification (GSI). North American Journal of Fisheries Management 34:184–201.

Hubisz, M. J., D. Falush, M. Stephens, and J. K. Pritchard. 2009. Inferring weak population structure with the assistance of sample group information. Molecular Ecology Resources 9:1322–1332.

Jensen, Ø., T. Dempster, E. B. Thorstad, I. Uglem, A. Fredheim, and others. 2010. Escapes of fishes from Norwegian sea-cage aquaculture: causes, consequences and prevention. Aquaculture Environment Interactions 1:71–83.

Kalinowski, S., K. Manlove, and M. Taper. 2007. ONCOR: a computer program for genetic stock identification. Department of Ecology, Montana State University, USA.

Kinnison, M. T., M. J. Unwin, and T. P. Quinn. 2008. Eco-evolutionary vs. habitat contributions to invasion in salmon: experimental evaluation in the wild. Molecular Ecology 17:405–414.

Kinnison, M. T., M. J. Unwin, and T. P. Quinn. 2003. Migratory costs and contemporary evolution of reproductive allocation in male chinook salmon. Journal of Evolutionary Biology 16:1257–1269.

Lindbergh, J. M. 1982. A successful transplant of Pacific salmon to Chile. Proceedings of the Gulf and Caribbean Fisheries Institute 34:81–87.

Lindbergh, J. M., R. E. Noble, and K. M. Blackburn. 1981a. Salmon ranching in Chile: the private sector. ICLARM newsletter.

Lindbergh, J., R. Noble, and K. Blackburn. 1981b. First returns of Pacific salmon to Chile.

Lucek, K., D. Roy, E. Bezault, A. Sivasundar, and O. Seehausen. 2010. Hybridization between distant lineages increases adaptive variation during a biological invasion: stickleback in Switzerland. Molecular Ecology 19:3995–4011.

McDowall, R. M. 1994. The origins of New Zealand's Chinook salmon, *Oncorhynchus tshawytscha*. Marine Fisheries Review 56:1–7.

Méndez, R., and C. Munita. 1989. La salmonicultura en Chile. Primera edición. Fundación Chile, Santiago.

Moran, P., J. F. Bromaghin, and M. Masuda. 2014. Use of genetic data to infer population-specific ecological and phenotypic traits from mixed aggregations. PLoS ONE 9:e98470.

Moran, P., D. J. Teel, M. A. Banks, T. D. Beacham, M. R. Bellinger, S. M. Blankenship, J. R. Candy, J. C. Garza, J. E. Hess, S. R. Narum, L. W. Seeb, W. D. Templin, C. G. Wallace, and C. T. Smith. 2013. Divergent life-history races do not represent Chinook salmon coast-wide: the importance of

scale in Quaternary biogeography. Canadian Journal of Fisheries and Aquatic Sciences 70:415–435.

Naylor, R. L., K. Hindar, I. A. Fleming, R. Goldburg, S. L. Williams, J. Volpe, F. Whoriskey, J. Eagle, D. Kelso, and M. Mangel. 2005. Fugitive salmon: assessing the risks of escaped fish from net-pen aquaculture. BioScience 55:427–437.

Peck, J. W., T. S. Jones, W. R. MacCallum, and S. T. Schram. 1999. Contribution of hatchery-reared fish to Chinook salmon populations and sport fisheries in Lake Superior. North American Journal of Fisheries Management 19:155–164.

Pritchard, J. K., M. Stephens, and P. Donnelly. 2000. Inference of population structure using multilocus genotype data. Genetics 155:945–959.

Putman, N. F., M. M. Scanlan, E. J. Billman, J. P. O'Neil, R. B. Couture, T. P. Quinn, K. J. Lohmann, and D. L. G. Noakes. 2014. An inherited magnetic map guides ocean navigation in juvenile Pacific salmon. Current Biology 24:446–450.

Quinn, T. P., M. T. Kinnison, and M. J. Unwin. 2001. Evolution of chinook salmon (*Oncorhynchus tshawytscha*) populations in New Zealand: pattern, rate, and process. Genetica 112-113:493–513.

Quinn, T. P., J. L. Nielsen, C. Gan, M. J. Unwin, R. Wilmot, C. Guthrie, and F. M. Utter. 1996. Origin and genetic structure of Chinook salmon, *Oncorhynchus tshawytscha*, transplanted from California to New Zealand: allozyme and mtDNA evidence. Fishery Bulletin 94:506–521.

Quinn, T. P., J. A. Peterson, V. F. Gallucci, W. K. Hershberger, and E. L. Brannon. 2002. Artificial selection and environmental change: countervailing factors affecting the timing of spawning by coho and Chinook salmon. Transactions of the American Fisheries Society 131:591–598.

Rannala, B., and J. L. Mountain. 1997. Detecting immigration by using multilocus genotypes. PNAS 94:9197–9201.

Rius, M., and J. A. Darling. 2014. How important is intraspecific genetic admixture to the success of colonising populations? Trends in Ecology & Evolution 29:233–242.

Riva-Rossi, C. M., M. A. Pascual, E. A. Marchant, N. Basso, J. E. Ciancio, B. Mezga, D. A. Fernández, and B. Ernst-Elizalde. 2012. The invasion of Patagonia by Chinook salmon (*Oncorhynchus tshawytscha*): inferences from mitochondrial DNA patterns. Genetica 140:439–453.

Roman, J. 2006. Diluting the founder effect: cryptic invasions expand a marine invader's range. Proc. R. Soc. B 273:2453–2459.

Sakai, M. 1989. Final report of aquaculture project in Chile. Japan International Cooperation Agency (JICA).

Seeb, L. W., A. Antonovich, M. A. Banks, T. D. Beacham, M. R. Bellinger, S. M. Blankenship, M. R. Campbell, N. A. Decovich, J. C. Garza, C. M. Guthrie III, T. A. Lundrigan, P. Moran, S. R. Narum, J. J. Stephenson, K. J. Supernault, D. J. Teel, W. D. Templin, J. K. Wenburg, S. F. Young, and C. T. Smith. 2007. Development of a standardized DNA database for Chinook salmon. Fisheries 32:540–552.

Sepúlveda, M., I. Arismendi, D. Soto, F. Jara, and F. Farias. 2013. Escaped farmed salmon and trout in Chile: incidence, impacts, and the need for an ecosystem view. Aquaculture Environment Interactions 4:273–283.

Snyder, B. P. 1971. Supplemental report on inland fresh water resources of central Chile.

Soto, D., F. Jara, and C. Moreno. 2001. Escaped salmon in the inner seas, southern Chile: facing ecological and social conflicts. Ecological Applications 11:1750–1762.

United Nations. 2006. Transfer of technology for successful integration into the global economy; A case study of the salmon industry in Chile. *in* United Nations conference on trade and development. United Nations.

Unwin, M. J. 2006. Assessment of significant salmon spawning sites in the Canterbury region. NIWA Client Report CHC2006-097. Unpublished report prepared for Environment Canterbury, Christchurch, New Zealand.

Urban, M. C., and L. De Meester. 2009. Community monopolization: local adaptation enhances priority effects in an evolving metacommunity. Proceedings of the Royal Society B: Biological Sciences 276:4129–4138.

Voisin, M., C. R. Engel, and F. Viard. 2005. Differential shuffling of native genetic diversity across introduced regions in a brown alga: Aquaculture vs. maritime traffic effects. PNAS 102:5432–5437.

Waters, J. M., C. I. Fraser, and G. M. Hewitt. 2013. Founder takes all: density-dependent processes structure biodiversity. Trends in Ecology & Evolution 28:78–85.

Zalewski, A., A. Michalska-Parda, M. Bartoszewicz, M. Kozakiewicz, and M. Brzeziński. 2010. Multiple introductions determine the genetic structure of an invasive species population: American mink *Neovison vison* in Poland. Biological Conservation 143:1355–1363.

Table 1: Chinook salmon deliberate and accidental releases in Patagonia since 1970 (a complete review spanning earlier introductions is available in Supporting Information, Table S1; modified from Correa and Gross 2008).

**Country year(s) Basin, Latitude River stocked**	**Number of Individuals Released**	**Ontogenetic Stage**	**Stock Origin**	**Adult Returns**	**Comments**
Chile 1970, 1971<br>Bueno, 40°S<br>Río Chirri[1]	50,000 (1970)[1]<br>270,150 (1971)[1]	Subyearlings (8mo, 15g, 12cm in 1970; 5mo, 5.5-17g, 6-12cm in 1971)[1]	USA; Green River Hatchery[1] (Cowlitz River, lower Columbia River, Washington).	?	Agriculture and Livestock Service of the Government of Chile and the U.S. Peace Corps. First shipment by plane[1,2]. Eggs were received at Lautaro Hatchery on December; stockings took place 36 (Sep 1970) or 23 weeks later (May 1971)[1].
Chile, 1978<br>Coastal, 42°S<br>Chiloé Island,<br>Curaco de Vélez[3,4,5,6]	120,000 (late 1978) [4,5]<br>170,000[3,6]	Smolts (1+; 70g)[4,5]	USA; Cowlitz River (lower Columbia River, Washington)[4,5,6]	Yes[4,5]	Domsea Pesquera Chile Ltd. (Union Carbide Corporation, USA), began salmon ocean-ranching experimentation[3,5,6]. In 1979, 334 returning jacks and 2 females were trapped[4,5].
Chile, 1979<br>Coastal, 42°S<br>Chiloé Island,<br>Curaco de Vélez[5]	190,000[4,5]	Smolts (1+)[4,5]	Idem[4,5]	Yes[4,5]	Domsea Pesquera Chile Ltd.[4,5].
Chile,1980-1982<br>Same location[6]	40,000-90,000[7]	Smolts (1+)[5]	Idem (?);<br>Progeny from local returnees[3, 6]	Yes[4,5,6]	In 1981, Domsea Pesquera Chile Ltd. was sold to Fundación Chile (private, non-profit), and renamed Salmones Antártica Ltd.[3,6]. Through july 1981, 817 adults had returned to the hatchery from previous brood years[4,5].
Chile, 1982<br>Coastal, 54°S<br>Río Santa María[6,8]	200,000[8]	Smolts[8]	University of Washington's stock [6,9];<br>Progeny from returnees at Curaco de Vélez[6,9] (?)	?[6,8]<br>Yes[10]	Fundación Chile through Salmones Antártica Ltd. launched new facility in the Magellan region subsequently destroyed by storm, and abandoned[6]. Jacks seen returning in 1983[8].
Chile, 1983<br>Prat, 51°S<br>Río Prat[11]	5,000[11]	Smolts (1+)[11]	USA[11]; University of Washington's stock[9,12] (?);<br>Progeny from returnees at Curaco de Vélez and Astilleros, Chiloé[12].	Yes (~2.3%)[11]	Fundación Chile through Salmones Antártica Ltd. launched another facility in the Magellan region with successfully returning spawners[6,8].
Chile,1987<br>Same location[11]	294,967[11] (USA origin)<br>40,042[11] (Chilean origin)	Smolts (1+)[11]	USA[11], seemingly University of Washington's[9];<br>Progeny from Chilean returnees[11] at Río Prat (local), Curaco de Vélez, and Astilleros[12,13]	Yes (~0.07% until 1989)[11]	Continuation of the above enterprise. In 1998 Fundación Chile and Salmones Antártica created Salmotec S.A.[14].

Chile, 1989,1990, 1993 Bueno 40°S Estero Huillín[15]	? (1989-1990) 3347 (Jan 1993)[15]	Smolts (<1)	? (1989-1990); Progeny from 1992 returning adults (38 females + 12 males)[15]	Yes, at least from 1989-1990 stockings[15]	Universidad de Los Lagos' experimentation at Piscicultura Experimental Lago Ranco[15].
Chile, 1987-2000 Coast, 39-45°S Inner seas[16]	100,000[17]	Mostly subadults[18]	Washington Sate[19,20] Oregon State[21] Vancouver Island[22] New Zealand[23] Alaska[24] (?) Australia[25] (?)	Yes[26]	Chinook salmon stocks were imported primarily to the Lakes District Region for net-pen culture. Last recorded importation in 2000.

Notes and references: The actual number of individuals released may be less than the figure reported due to mortality during transport and handling; pre-release mortality was accounted for whenever possible. Approximate latitude is given at the river mouth. [? = unreported, likely stock origin, or lack of adults return assessment]; [1] Snyder 1971; [2] Ellis and Salo (1969) in Basulto (2003); [3] Fundación Chile (1990); [4] Lindbergh et al. (1981); [5] Lindbergh (1982); [6] Méndez and Munita (1989); [7] Estimate subtracting the above 1978-1979 stockings from c. 400,000 stocked in the period 1978-1982 (Méndez and Munita 1989, P. 45). Subsequent releases (1983-1989) at the same location and at a nearby company's new facility (Piscicultura Astilleros) might have also included Chinook salmon (Méndez and Munita 1989, P. 164).; [8] Basulto (2003); [9] Donaldson and Joyner (1983); [10] Manuel Barros personal communication (2008) in (Aedo 2011). At the time, M. Barros worked for Fundación Chile.; [11] Salmotec Ltd. in Sakai (1989); [12] Cristian Jélvez personal communication (2005) in (Aedo 2011). C. Jélvez worked for Fundación Chile (1982). In addition, we found commercial invoices and fish transfer records of two shipments of fall-run eyed eggs (280,000 and 320,000 eggs, respectively) shipped from University of Washington Hatchery to Fundación Chile in December 1981; [13] Fredy Carrasco personal communication (2005) in (Aedo 2011). F. Carrasco worked for Fundación Chile (1986).; [14] United Nations (2006); [15] Del Real (1993). Aedo (2011) mentioned other stocking locations (Río Contaco and Río Maicolpué) by Universidad de los Lagos, but we found no further records of these; [16] Primarily marine aquaculture concessions in the Lake District region.; [17] Rough estimate of number of sub-adult Chinook salmon escapees (see main text). [17] Mostly 1+ year class and older since most escapes were from marine net-pens (Soto et al. 2001).; [19] Follow fragmentary records of ova imported (OI) by the Chilean aquaculture industry in 1987-2000 (Aedo 2011). Some information of providers was available for 60% of the imports; we report specific lineages and origins of livestock whenever possible, and ova suppliers and/or geographic origin of shipments otherwise. Additional potential sources of the unaccounted imports were identified from import permits (OP) issued by the Chilean National Fisheries Service (SERNAPESCA), although it remains unclear if these planed importations ever materialized. Sources listed in decreasing order of importance (Aedo 2011); [20] OI: Columbia River. OP: Fish Pro Inc. and University of Washington; [21] OI: Springfield. OP: Aqua Food, Aquafoods, and Aqua Seed Corp.; [21] OI: Koksilah River. OP: Sea Spring Salmon Farms Ltd., Hardy Sea Farms, Hadfield Consultants Inc., Hatfield International SA., Fishpro, and Aqua Seed; [23] OI: Sanford Waitaki Salmon Hatchery (Kaitan Gata). OP: Big Glory Bay Hatchery, and Kaitan Gata Hatchery and Sanford Waitaki Salmon Hatchery (Stewart Island).; [24] OP: Sitka; [25] OP: Tasmania; [26] This study.

Table 2: Genetic ancestral contribution of North American lineages to Patagonian Chinook salmon populations based on reporting group-level CML mixture analysis.

		Patagonian population				
Id	Reporting group	Toltén	Petrohué	Aysén	Baker	Pooled
1	Central Valley fa		8.2 (2)			2.4 (2)
5	Klamath R	6.9 (1)				1.3 (1)
6	Chetco R	7.3 (1)				1.3 (1)
9	Willamette R sp			5.3 (1)	18.3 (5)	6.6 (6)
13	N Oregon Coast	9.5 (1)	8.4 (2)		0.5 (0)	4.3 (3)
15	W Cascade fa	9.5 (2)	3.8 (1)	17.3 (3)	10.9 (3)	10.1 (9)
16	W Cascade sp	7.3 (1)	21.7 (5)	71.3 (15)	64.5 (15)	43.4 (36)
17	Interior Columbia Basin su/fa	19 (3)	9.0 (2)	4.4 (1)	5.6 (1)	8.7 (7)
19	S Puget Sound fa	0.2 (0)	20.0 (5)	1.3 (0)	0.1 (0)	6.2 (5)
22	Washington Coast	6.5 (1)	8.0 (2)	0.2 (0)		3.6 (3)
23	Straits of Juan de Fuca	0.2 (0)	5.7 (1)			1.7 (1)
24	Whidbey Basin	20.1 (3)	4.5 (1)	0.2 (0)		5 (4)
26	E Vancouver Is	6.3 (1)	2.2 (1)			1.8 (2)
31	S Thompson R	6.4 (1)	0.1 (0)			1.2 (1)
38	SSE Alaska	0.6 (0)	4.4 (1)			1.4 (1)
39	Nass R		3.9 (1)			1.1 (1)
	No. individuals in mixture	(15)	(24)	(20)	(24)	(83)

Notes: Patagonian populations are ordered from north to south, and the last column represents all populations pooled. Values represent average percent genetic contribution; in brackets, frequency of individual assignments to baseline reporting groups, as inferred from individual's highest assignment probability. Populations within baseline reporting groups were pooled (for population-level mixture analysis, see Supporting Information, Table S2). Contributions >= 10% were highlighted with boldface (see text). Identifiers (Id) correspond to those in Figure 1. Reporting groups receiving assignment probabilities ~ 0 were omitted.

Figure captions

Figure 1: Chinook salmon native (green) and invasive (brown) range in the Americas, and results of reporting group-level CML mixture analysis (connecting vectors). The North American continent has been rotated and translated such that the North American and South American Pacific coasts face each other matching latitudes (longitudes were shifted for clarity). Connecting vectors represent average genetic contribution (proportional to line thickness) of the 46 North American baseline reporting groups used in CML mixture analysis. Reporting groups were characterized by 146 baseline populations (dots).

Figure 2: Distribution of individual maximum assignment probabilities from reporting group-level CML mixture analysis. High assignment probabilities were observed even in Toltén and Petrohué where many lineages occurred in sympatry.

Figure 3: Model-based clustering showed the clearest discrimination among the 31 baseline populations at K = 5. These five clusters (Q1 to Q5 in panel A) were highly concordant with the seven reporting groups containing baseline populations (number of populations per reporting group in parenthesis). Patagonian samples were treated as having unknown origin, and showed a spatially heterogeneous pattern of membership to the five clusters (B), broadly concordant with CML analysis.

Figure 1

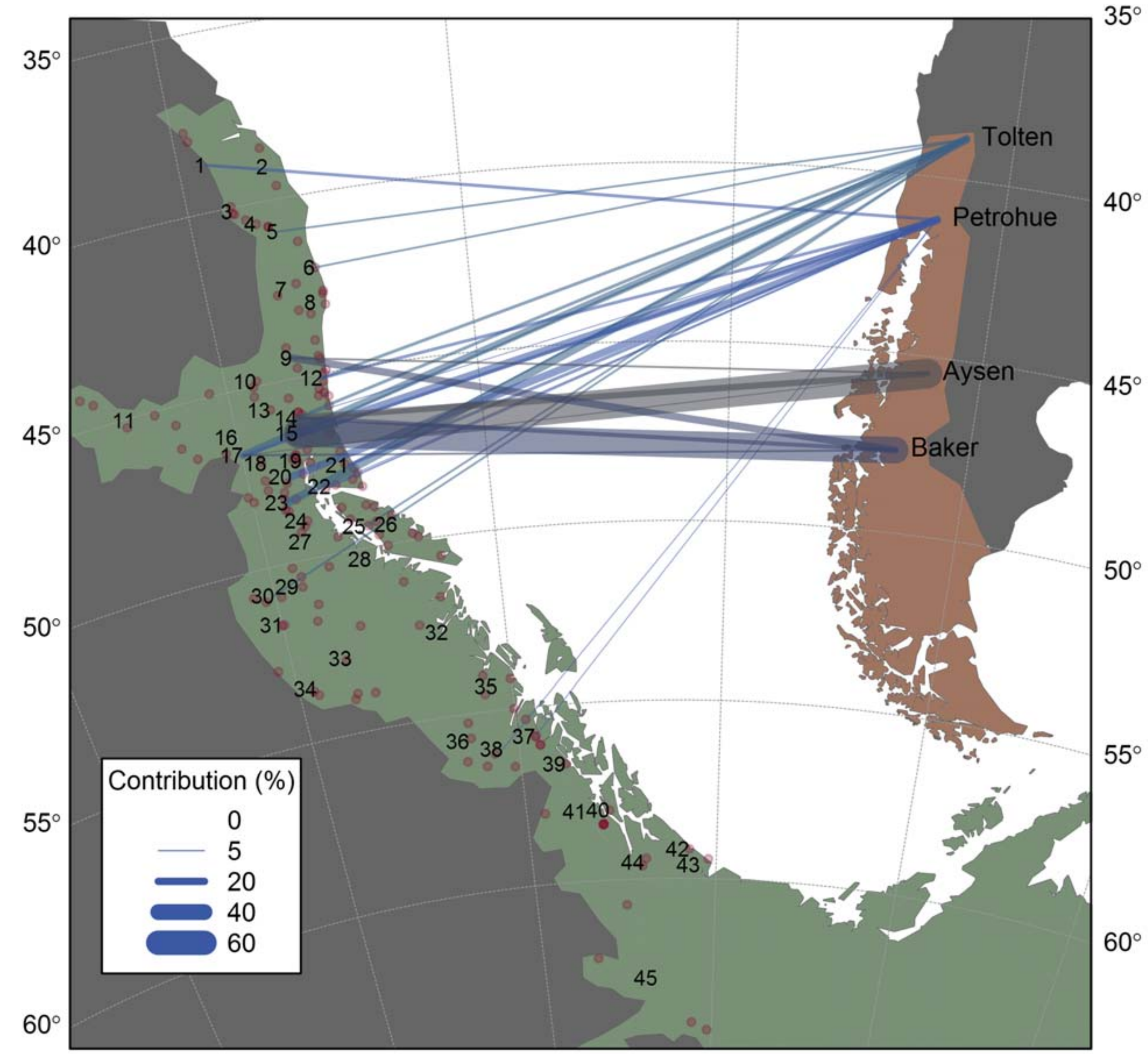

GAPS Reporting groups

1 Central Valley fa
2 California Coast
3 Central Valley sp
4 Central Valley wi
5 Klamath R
6 Chetco R
7 Rogue R
8 Mid Oregon Coast
9 Willamette R sp
10 Deschutes R sp
11 Snake R sp/su
12 N Oregon Coast
13 Spring Cr Group tule
14 W Cascade fa
15 W Cascade sp
16 Mid and Upper Columbia sp
17 Interior Columbia Basin su/fa
18 Yakima R
19 S Puget Sound sp
20 S Puget Sound fa
21 Washington Coast
22 Straits of Juan de Fuca
23 Whidbey Basin
24 Nooksack R
25 E Vancouver Is
26 W Vancouver Is
27 L Fraser R
28 S BC Mainland
29 L Thompson R
30 S Thompson R
31 N Thompson R
32 Central BC Coast
33 Mid Fraser R
34 U Fraser R
35 L Skeena R
36 U Skeena R
37 SSE Alaska
38 Nass R
39 SSE Alaska Stikine R
40 NSE Alaska King Salmon R
41 Taku R
42 N Gulf Coast Alsek R
43 N Gulf Coast Situk R
44 NSE Alaska Chilkat R
45 Yukon

Figure 2

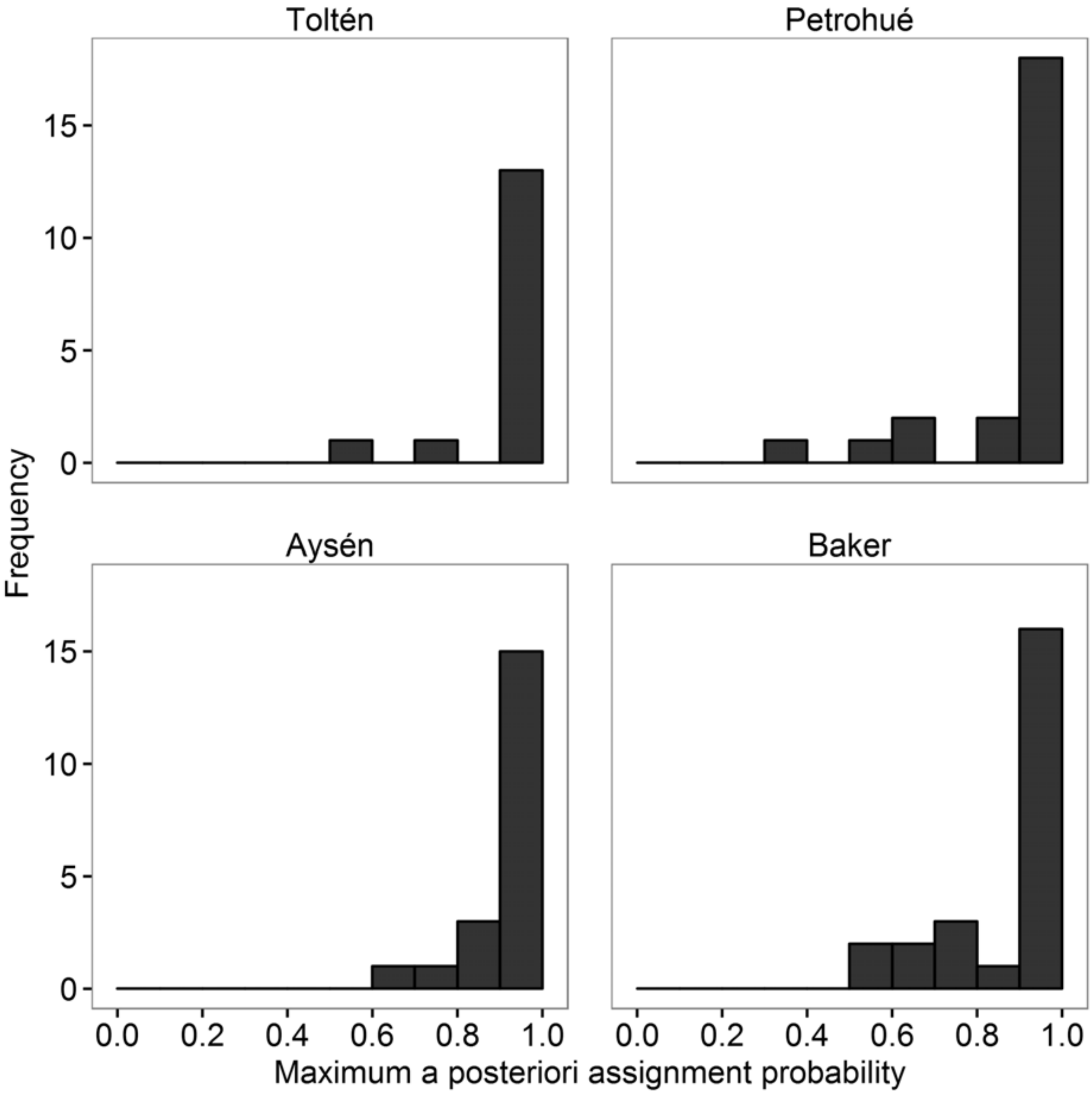
Toltén
Petrohué
Aysén
Baker
Frequency
0
5
10
15
0.0
0.2
0.4
0.6
0.8
1.0
Maximum a posteriori assignment probability

Figure 3

(A)

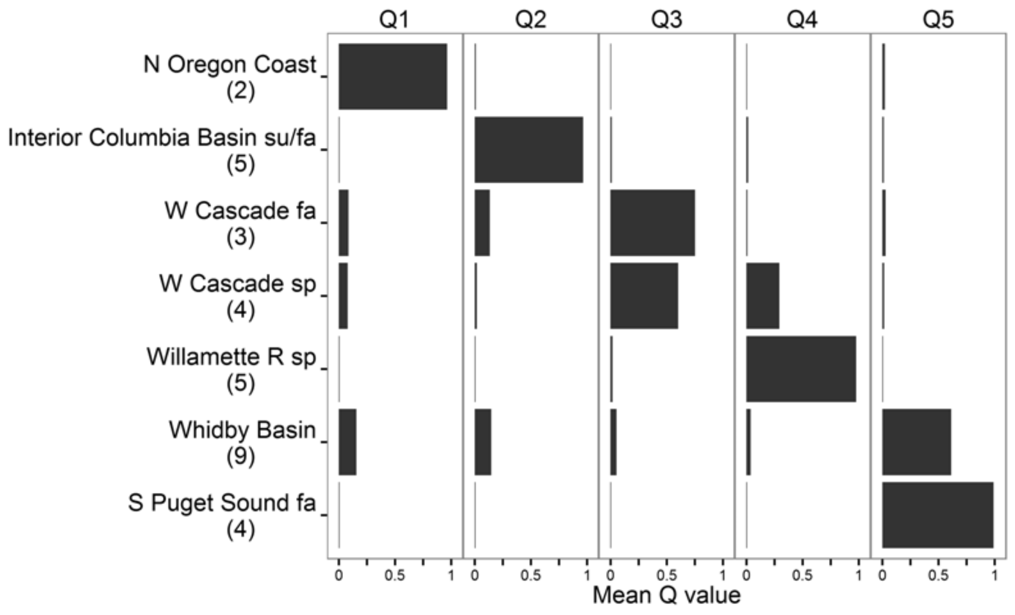
Q1
Q2
Q3
Q4
Q5
N Oregon Coast (2)
Interior Columbia Basin su/fa (5)
W Cascade fa (3)
W Cascade sp (4)
Willamette R sp (5)
Whidby Basin (9)
S Puget Sound fa (4)
0
0.5
1
Mean Q value

(B)

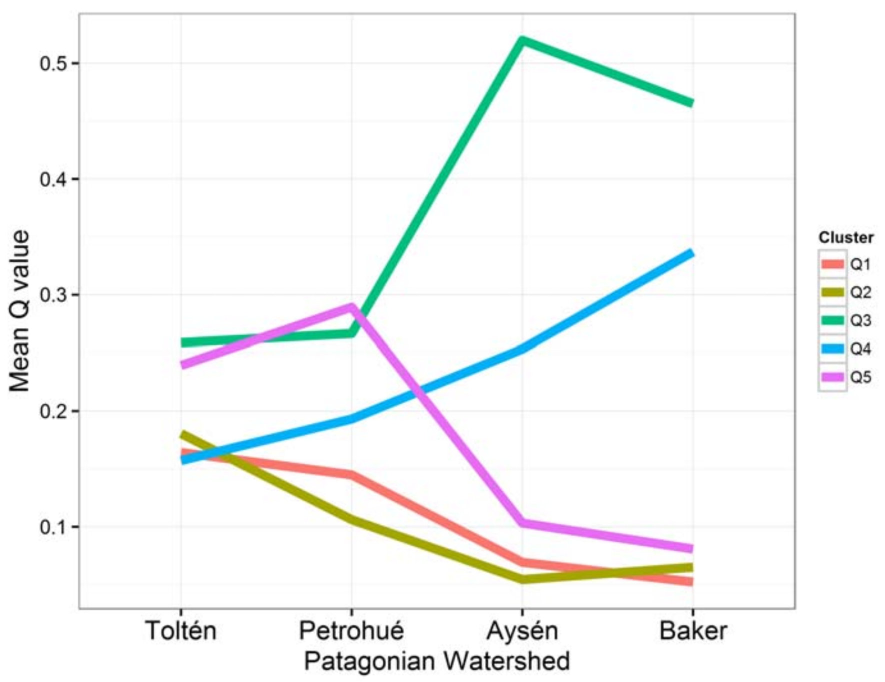
0.5
0.4
0.3
0.2
0.1
Mean Q value
Toltén
Petrohué
Aysén
Baker
Patagonian Watershed
Cluster
Q1
Q2
Q3
Q4
Q5

Supporting Information

Additional Supporting Information may be found in the online version of this article at the publisher's website:

Table S1: Full review of Chinook salmon deliberate and accidental releases in Latin America.

Table S2: Genetic ancestral contribution of North American lineages to Patagonian Chinook salmon based on population-level CML mixture analysis.

Figure S1: Distribution of individual maximum assignment probabilities from population-level CML mixture analysis.

Figure S2: CML mixture analysis of simulated mixed-origin Chinook salmon population.

Table S1: Chinook salmon deliberate and accidental releases in Latin America (modified from Correa and Gross 2008).

Country year(s) Basin, Latitude River stocked	Number of Individuals Released	Ontogenetic Stage	Stock Origin	Adult Returns	Comments
Mexico 1891-1900<br>?	50,000[1]	?	USA[2]; Sacramento River (?)[1]	No[2, 1]	"From [1872-1930] the [US] Bureau of Fisheries, with benevolent intent, supplied over 100 million eggs of Pacific salmon (Chinook) to people in other countries, with the idea of establishing new salmon runs there—a considerable attempt to bring in the New World to right the Rest."[3]
Mexico 1901-1910<br>?	50,000[1]	?	USA[2]; Sacramento River (?)[1]	No[2, 1]	
Nicaragua 1901-1910<br>?	20,000[1]	?	USA[2]; Sacramento River (?)[1]	No[2, 1]	
Argentina 1906<br>Santa Cruz, 50°S[4]<br>Gallegos, 52°S[4]	300,000[4]	?	USA; Sacramento River (?)[4]	No (?)[5]	
Argentina 1908<br>Chico, 50°S[6]<br>Santa Cruz, 50°S[6]	300,000[6]	?	USA; Sacramento River (?)[6]	No (?)[5]	
Argentina 1909<br>Chico, 50°S[6]<br>Santa Cruz, 50°S[6]	200,000[6]	?	USA; Sacramento River (?)[6]	No (?)[5]	
Argentina 1910<br>(Rivers of Santa Cruz Province[6])	200,000[6]	?	USA; Sacramento River (?)[6]	No (?)[5]	
Argentina 1901-1910[1]<br>?	1,058,000[1]	?	USA[2]; Sacramento River (?)[1]	No?[2, 5]	The last Argentinean entry (1901-1910) might include the previous four.
Brazil, 1958<br>Jaquarí, 30°S<br>Río Caí[7]<br>RíoTainhos[7]<br>Río dos Antos[7]	400,000[7]	Fertilized eggs[7]	USA; American River, California[7]	No (?)[7]	Although there were no reports of salmon returning to the Río Jaquarí, large fish of a species unknown to local residents were seen leaping falls in the Río Uruguay in 1962[7].
Chile, 1924<br>Imperial, 39°S<br>Río Cautin8[, 9]<br>Maullin,42°S<br>Río Maullín[8, 9]<br>Cochamó, 42°S<br>Río Cochamó[9]<br>Puelo,42°S<br>Río Puelo[8, 9]	200,000[9,10] (little less than 50% died during transport[9])	Fingerlings (4mo[9])	USA; Sacramento River; McCloud River Hatchery (?)[10]	No (?)[8,9,10]	The U.S. government presented the government of Chile with 200,000[10, 9] fertilized ChS eggs. The embryos arrived at a recently built hatchery in Rio Blanco (near Santiago) just prior to hatching[8, 9]. After four months, fingerlings were transported by rail and released[9].

Chile 1970, 1971<br>Bueno, 40°S<br>Río Chirri11	50,000 (1970)[11]<br>270,150 (1971)[11]	Subyearlings (8mo, 15g, 12cm in 1970; 5mo, 5.5-17g, 6-12cm in 1971)[11]	USA; Green River Hatchery[11] (Cowlitz River, lower Columbia River, Washington).	?	Agriculture and Livestock Service of the Government of Chile and the U.S. Peace Corps. First shipment by plane[11,12]. Eggs were received at Lautaro Hatchery on December; stockings took place 36 (Sep 1970) or 23 weeks later (May 1971)[11].
Chile, 1978<br>Coastal, 42°S<br>Chiloé Island,<br>Curaco de Vélez[10,13,14,15]	120,000<br>(late 1978) [13,14]<br>170,000[10,15]	Smolts (1+; 70g)[13,14]	USA; Cowlitz River (lower Columbia River, Washington)[13,14,15]	Yes[13,14]	Domsea Pesquera Chile Ltd. (Union Carbide Corporation, USA), began salmon ocean-ranching experimentation[10,14,15]. In 1979, 334 returning jacks and 2 females were trapped[13,14].
Chile, 1979<br>Coastal, 42°S<br>Chiloé Island,<br>Curaco de Vélez[14]	190,000[13,14]	Smolts (1+)[13,14]	Idem[13,14]	Yes[13,14]	Domsea Pesquera Chile Ltd.[13,14].
Chile,1980-1982<br>Same location[15]	40,000-90,00016	Smolts (1+)[14]	Idem (?);<br>Progeny from local returnees[10, 15]	Yes[13,14,15]	In 1981, Domsea Pesquera Chile Ltd. was sold to Fundación Chile (private, non-profit), and renamed Salmones Antártica Ltd.[10,15]. Through july 1981, 817 adults had returned to the hatchery from previous brood years[13,14].
Chile, 1982<br>Coastal, 54°S<br>Río Santa María[15,17]	200,000[17]	Smolts[17]	University of Washington's stock [15,18] (?);<br>Progeny from returnees at Curaco de Vélez[15,18] (?)	?[15,17]<br>Yes[19]	Fundación Chile through Salmones Antártica Ltd. launched new facility in the Magellan region subsequently destroyed by storm, and abandoned[15]. Jacks seen returning in 1983[17].
Chile, 1983<br>Prat, 51°S<br>Río Prat[20]	5,000[20]	Smolts (1+)[20]	USA[20]; University of Washington's stock[18,21] (?);<br>Progeny from returnees at Curaco de Vélez and Astilleros, Chiloé[21].	Yes (~2.3%)[20]	Fundación Chile through Salmones Antártica Ltd. launched another facility in the Magellan region with successfully returning spawners[15,17].
Chile,1987<br>Same location[20]	294,967[20]<br>(USA origin)<br>40,042[20]<br>(Chilean origin)	Smolts (1+)[20]	USA[20], seemingly University of Washington's[18];<br>Progeny from Chilean returnees[20] at Río Prat (local), Curaco de Vélez, and Astilleros[21,22]	Yes (~0.07% until 1989)[20]	Continuation of the above enterprise. In 1998 Fundación Chile and Salmones Antártica created Salmotec S.A.[23].
Chile, 1989,1990, 1993<br>Bueno 40°S<br>Estero Huillín[24]	? (1989-1990)<br>3347 (Jan 1993)[24]	Subyearling smolts (1993)[24]	? (1989-1990);<br>Progeny from 1992 returning adults (38 females + 12 males)[24]	Yes, at least from 1989-1990 stockings[24]	Universidad de Los Lagos' experimentation at Piscicultura Experimental Lago Ranco[24].

Chile, 1987-2000<br>Coast, 39-45°S<br>Inner seas[25]	100,000[26]	Mostly subadults[27]	Washington Sate[28,29]<br>Oregon State[30]<br>Vancouver Island[31]<br>New Zealand32<br>Alaska[33] (?)<br>Australia[34] (?)	Yes[35]	Chinook stocks were imported primarily to the Lakes District Region for commercial net pen rearing. Last recorded importation in 2000.

Notes and references: The actual number of individuals released may be less than the figure reported due to mortality during transport and handling; pre-release mortality was accounted for whenever possible. Approximate latitude is given at the river mouth. ? = unreported, likely stock of origin, or lack of assessment of adult returns; [1] Davidson and Hutchinson (1938); [2] Welcomme (1988); [3] Elton (1958); [4] Tulian (1908) in Ciancio et al. (2005); [5] Marini (1936) in Davidson and Hutchinson (1938); [6] Marini and Mastrarrigo (1963) in Ciancio et al. (2005); [7] Joyner (1980); [8] Golusda (1927); [9] Barros (1931); [10] Fundación Chile (1990); [11] Snyder (1971); [12] Ellis and Salo (1969) in Basulto (2003); [13] Lindbergh et al. (1981); [14] Lindbergh (1982); [15] Méndez and Munita (1989); [16] Estimate subtracting the above 1978-1979 stockings from c. 400,000 stocked in the period 1978-1982 (Méndez and Munita 1989, P. 45). Subsequent releases (1983-1989) at the same location and at a nearby company's new facility (Piscicultura Astilleros) might have also included Chinook salmon (Méndez and Munita 1989, P. 164); [17] Basulto (2003); [18] Donaldson and Joyner (1983); [19] Manuel Barros, personal communication (2008) in Aedo (2011). At the time, M. Barros worked for Fundación Chile; [20] Salmotec Ltd. in Sakai (1989); [21] Cristian Jélvez, personal communication (2005) in Aedo (2011). C. Jélvez worked for Fundación Chile (1982). In addition, commercial invoices and fish transfer records indicated two shipments of fall-run eyed eggs (280,000 and 320,000 eggs, respectively) shipped from University of Washington Hatchery to Fundación Chile in December, 1981; [22] Fredy Carrasco, personal communication (2005) in Aedo (2011). F. Carrasco worked for Fundación Chile (1986); [23] United Nations (2006); [24] Del Real (1993). Aedo (2011) mentioned other stocking locations (Río Contaco and Río Maicolpué) by the Universidad de los Lagos program, but we could not confirm these records; [25] Primarily marine aquaculture concessions in the Lake District region; [26] Rough estimate of number of sub-adult Chinook salmon escapees (see main text); [27] Mostly 1+ year class and older since most escapes were from marine net-pens (Soto et al. 2001); [28] Follow fragmentary records of ova imported (OI) by the Chilean aquaculture industry in 1987-2000 (Aedo 2011). Some information of providers was available for 60% of the imports. We report specific lineages and origins of livestock whenever possible, and ova suppliers and/or geographic origin of shipments otherwise. Additional potential sources of the unaccounted imports were identified from import permits (OP) issued by the Chilean National Fisheries Service (SERNAPESCA), although it remains unclear if these planed importations ever materialized. Sources listed in decreasing order of importance (Aedo 2011); [29] OI: Columbia River. OP: Fish Pro Inc. and University of Washington; [30] OI: Springfield. OP: Aqua Food, Aquafoods, and Aqua Seed Corp.; [31] OI: Koksilah River. OP: Sea Spring Salmon Farms Ltd., Hardy Sea Farms, Hadfield Consultants Inc., Hatfield International SA., Fishpro, and Aqua Seed; [32] OI: Sanford Waitaki Salmon Hatchery (Kaitan Gata). OP: Big Glory Bay Hatchery, and Kaitan Gata Hatchery and Sanford Waitaki Salmon Hatchery (Stewart Island).; [33] OP: Sitka; [34] OP: Tasmania; [35] This study.

References

Aedo, E. 2011. Información sobre siembras de salmónidos en el ambiente natural e incidencia de escapes desde centros de cultivo en la Región de Aysén, referenciada geográficamente. *In* Evaluación cuantitativa del estado trofico de salmonidos de vida libre en el fiordo Aysén, XI región. Informe final proyecto FIP200830. *Edited by* E. Niklitschek and P. Toledo. Universidad Austral de Chile - Subsecretaría de Pesca, Puerto Montt. pp. 120–135.

Barros, R. 1931. Introducción de un nuevo salmón en Chile. Revista Chilena de Historia Natural 35: 57–62.

Basulto, S. 2003. El largo viaje de los salmones. Una crónica olvidada. Propagación y cultivo de especies acuáticas en Chile. Maval Ltda., Santiago.

Ciancio, J.E., Pascual, M.A., Lancelotti, J., Rossi, C.M.R., and Botto, F. 2005. Natural colonization and establishment of a chinook salmon,

Oncorhynchus tshawytscha, population in the Santa Cruz River, an Atlantic basin of Patagonia. Environmental Biology of Fishes 74: 219–227.

Correa, C., and Gross, M.R. 2008. Chinook salmon invade southern South America. Biological Invasions 10: 615–639. doi: 10.1007/s10530-007-9157-2.

Davidson, F.A., and Hutchinson, S.J. 1938. The geographic distribution and environmental limitations of the Pacific salmon (genus *Onchorhynchus*). Bulletin of the Bureau of Fisheries 48: 667–692.

Del Real, A. 1993. Antecedentes sobre el cultivo de salmón Chinook (*Oncorhynchus tshawystcha* W.) en la fase de agua dulce proveniente de reproductores retornantes en la piscicultura experimental Lago Rupanco. Seminario (Ingeniería de Ejecución en Acuicultura), Universidad de Los Lagos, Departamento de Acuicultura y Recursos Acuaticos, Osorno, Chile.

Donaldson, L.R., and Joyner, T. 1983. The salmonid fishes as a natural livestock. Scientific American 249: 50–58.

Elton, C.S. 1958. The ecology of invasions by animals and plants. University of Chicago Press.

Fundación Chile. 1990. El libro del salmon. Fundación Chile, Santiago.

Golusda, P. 1927. Aclimatación y cultivo de especies salmonídeas en Chile. Boletin de la Sociedad de Biología de Concepción 1(1 y 2): 80–100.

Joyner, T. 1980. Salmon ranching in South America. *In* Salmon ranching. *Edited by* T. John E. Academic Press Inc., London, England.

Lindbergh, J.M. 1982. A successful transplant of Pacific salmon to Chile. Proceedings of the Gulf and Caribbean Fisheries Institute 34: 81–87.

Lindbergh, J.M., Noble, R.E., and Blackburn, K.M. 1981. Salmon ranching in Chile: the private sector. ICLARM newsletter.

Méndez, R., and Munita, C. 1989. La salmonicultura en Chile. *In* Primera edición. Fundación Chile, Santiago.

Sakai, M. 1989. Final report of aquaculture project in Chile. Japan International Cooperation Agency (JICA).

Snyder, B.P. 1971. Supplemental report on inland fresh water resources of central Chile. United Nations Development Program, Report UNDP/23/71. 35pp.

Soto, D., Jara, F., and Moreno, C. 2001. Escaped salmon in the inner seas, southern Chile: facing ecological and social conflicts. Ecological Applications 11(6): 1750–1762.

United Nations. 2006. Transfer of technology for successful integration into the global economy; A case study of the salmon industry in Chile. *In* United Nations conference on trade and development. United Nations.

Welcomme, R.L. 1988. International introductions of inland aquatic species. FAO Fisheries Technical Paper 294.

Table S2: Genetic ancestral contribution of North American lineages to Patagonian Chinook salmon based on population-level CML mixture analysis.

ID	Reporting Group	NA Population	Patagonian watershed				
			Toltén	Petrohué	Aysén	Baker	Pooled
1	Central Valley fa	Stanislaus R		4.1 (1)			1.2 (1)
1	Central Valley fa	Tuolumne R		4.1 (1)			1.2 (1)
5	Klamath R	Klamath R fa	6.9 (1)				1.3 (1)
6	Chetco R	Chetco R	7.3 (1)				1.3 (1)
9	Willamette R sp	N Santiam H			4 (1)	3.3 (1)	1.9 (2)
9	Willamette R sp	McKenzie H			1.3 (0)	15 (4)	4.6 (4)
13	N Oregon Coast	Salmon R f	0.6 (0)	4.1 (1)			1.3 (1)
13	N Oregon Coast	Siuslaw R	2.9 (0)	4.2 (1)		0.5 (0)	1.9 (1)
13	N Oregon Coast	Trask R	5.9 (1)	0.1 (0)			1.1 (1)
15	W Cascade fa	Cowlitz H fa	8.9 (2)	3.2 (1)	16.8 (3)	5.4 (1)	8.2 (7)
15	W Cascade fa	Sandy R	0.6 (0)	0.6 (0)	0.5 (0)	5.5 (2)	2 (2)
16	W Cascade sp	Kalama H sp	3 (0)	15.2 (4)	26.9 (7)	28.7 (7)	19.7 (18)
16	W Cascade sp	Cowlitz H sp	4.3 (1)	6.4 (1)	44.4 (8)	35.8 (8)	23.7 (18)
17	Interior Columbia Basin su/fa	Wenatchee R s/f	6.8 (1)	0.4 (0)			1.4 (1)
17	Interior Columbia Basin su/fa	Hanford Reach	6.2 (1)	8.5 (2)	4.4 (1)	5.5 (1)	6.2 (5)
17	Interior Columbia Basin su/fa	Lyons Ferry H	6 (1)	0.1 (0)		0.1 (0)	1.1 (1)
19	S Puget Sound fa	Clear Cr H	0.1 (0)	2.1 (0)	1.3 (0)		0.9 (0)
19	S Puget Sound fa	Soos H		16.9 (5)		0.1 (0)	4.9 (5)
19	S Puget Sound fa	S Prairie Cr	0.1 (0)	1 (0)			0.3 (0)
22	Washington Coast	Sol Duc H		4 (1)	0.1 (0)		1.2 (1)
22	Washington Coast	Forks Cr H	6.5 (1)	4 (1)	0.1 (0)		2.4 (2)
23	Straits of Juan de Fuca	Elwha R	0.2 (0)	5.7 (1)			1.7 (1)
24	Whidbey Basin	Suiattle R		4.2 (1)			1.2 (1)
24	Whidbey Basin	Cascade R U	20.1 (3)	0.3 (0)	0.2 (0)		3.8 (3)
26	E Vancouver Is	Big Qual H	6.3 (1)	2.2 (1)			1.8 (2)
31	S Thompson R	L Adams H	6.4 (1)	0.1 (0)			1.2 (1)
38	SSE Alaska	Clear Cr	0.6 (0)	4.4 (1)			1.4 (1)
39	Nass R	Kincolith R		3.9 (1)			1.1 (1)
	No. Individuals in the mixture		(15)	(24)	(20)	(24)	(83)

Notes: Values represent average percent genetic contribution; in brackets, frequency of individual assignments to baseline populations, as

inferred from individual's highest assignment probability. Identifiers (ID) correspond to those in Figure 1 (main article).

Figure S1: Distribution of individual maximum assignment probabilities from population-level CML mixture analysis.

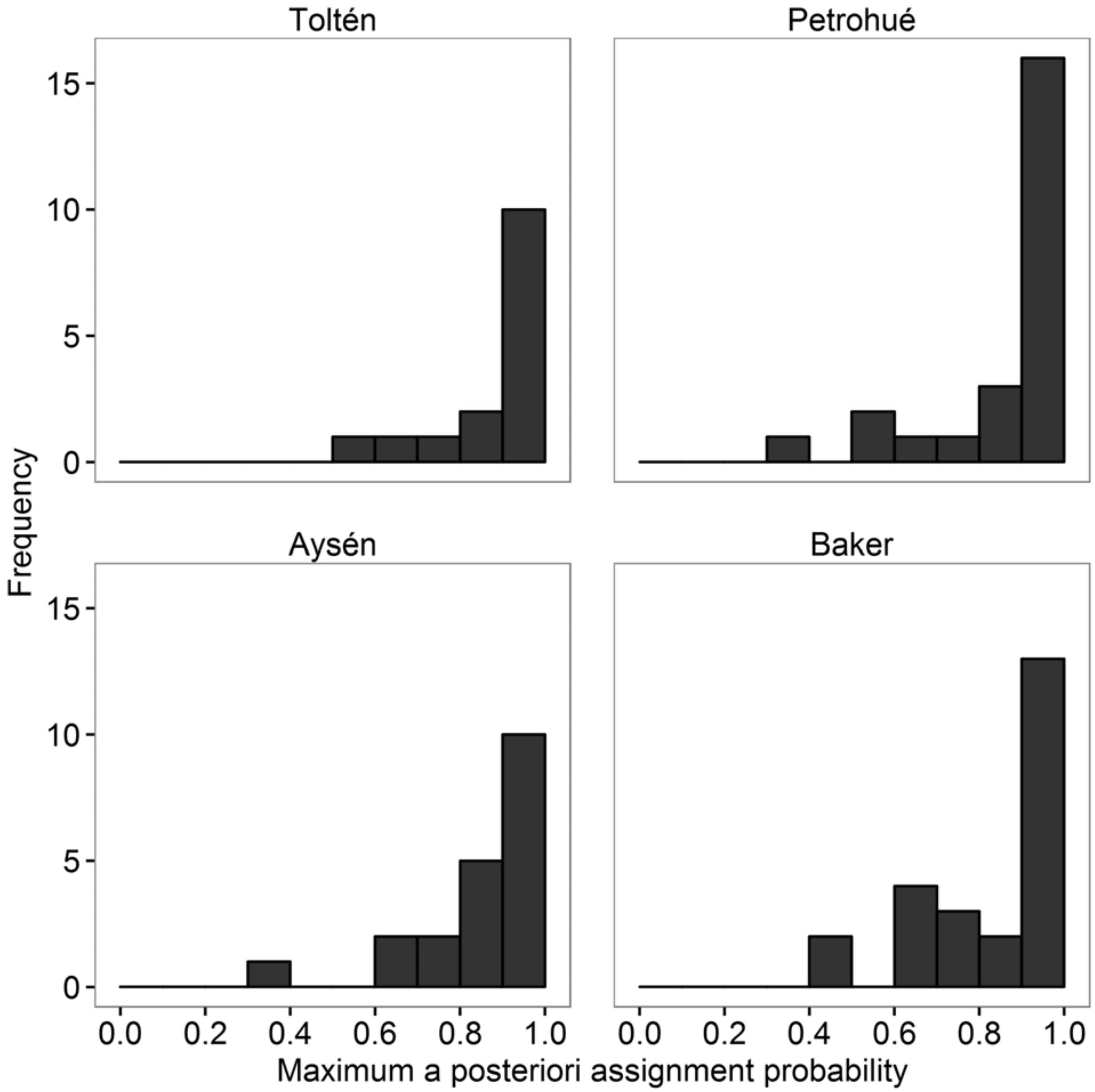

Figure S2: Conditional maximum likelihood (CML) mixture analysis of the simulated mixed-origin Chinook salmon population derived from Cowlitz River Hatchery spring run in the West Cascade spring-run reporting group and Soos Creek Hatchery fall run in the South Puget Sound fall-run reporting group. Distribution of maximum assignment probabilities (a), average percent genetic contribution of reporting groups to the simulated population (b), and individual alternative assignments based on best and second-best assignment probabilities (c). Equivocal assignments [i.e., low assignment probability, symbolized with darker lines in (c)] typically split probabilities between founder lineages, or between founder lineages and genetically similar reporting groups (see main text). A small fraction of simulated individuals assigned to unrelated lineages, even with high assignment probabilities in some cases. Reporting groups with no assignments were omitted. Reporting groups were ordered by decreasing order of estimated contribution (b) or increasing latitude (c).

(A)

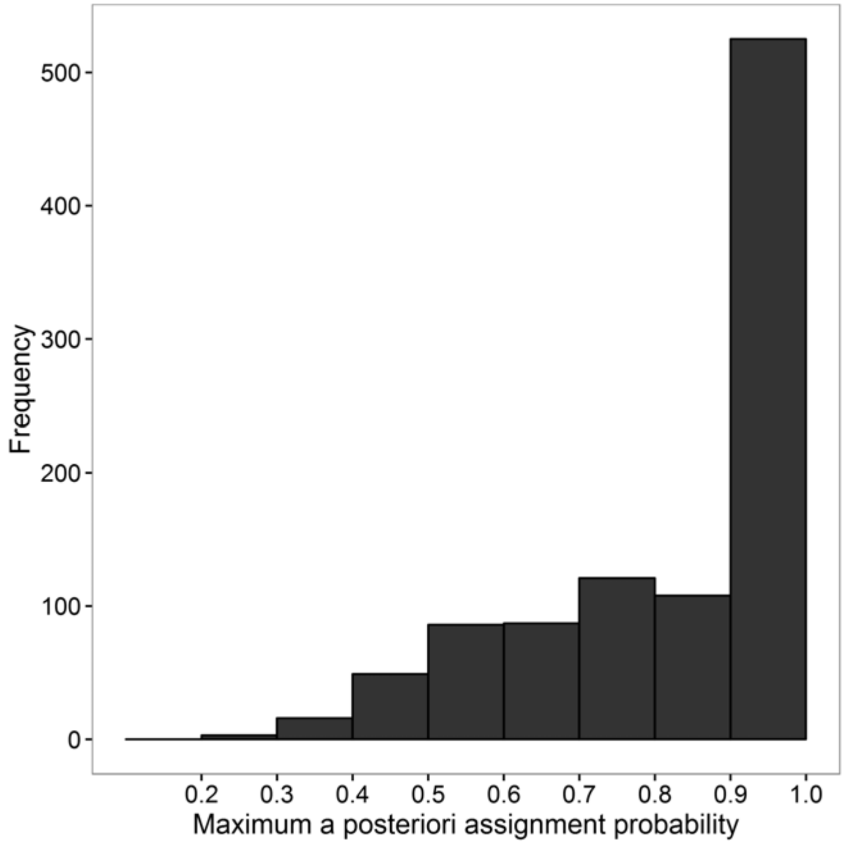

(B)

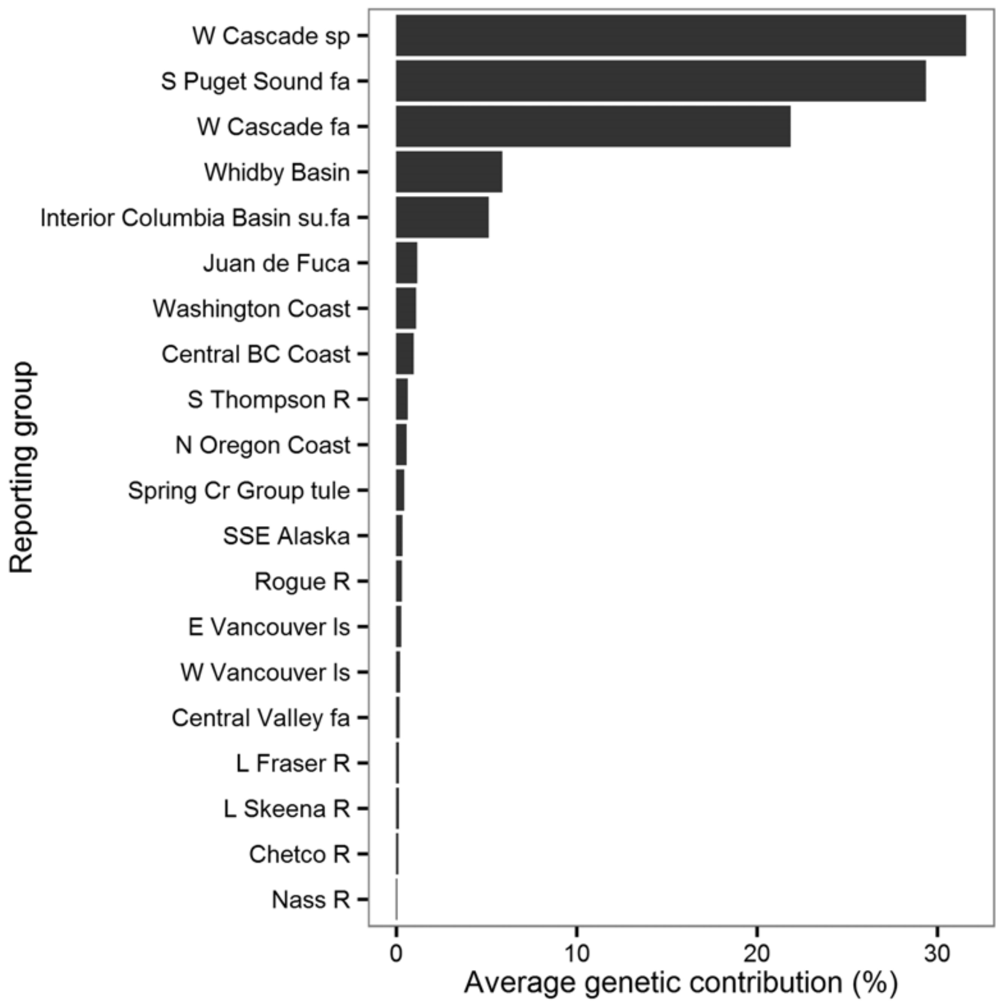
W Cascade sp
S Puget Sound fa
W Cascade fa
Whidby Basin
Interior Columbia Basin su.fa
Juan de Fuca
Washington Coast
Central BC Coast
S Thompson R
N Oregon Coast
Spring Cr Group tule
SSE Alaska
Rogue R
E Vancouver Is
W Vancouver Is
Central Valley fa
L Fraser R
L Skeena R
Chetco R
Nass R
Reporting group
0
10
20
30
Average genetic contribution (%)

(C)

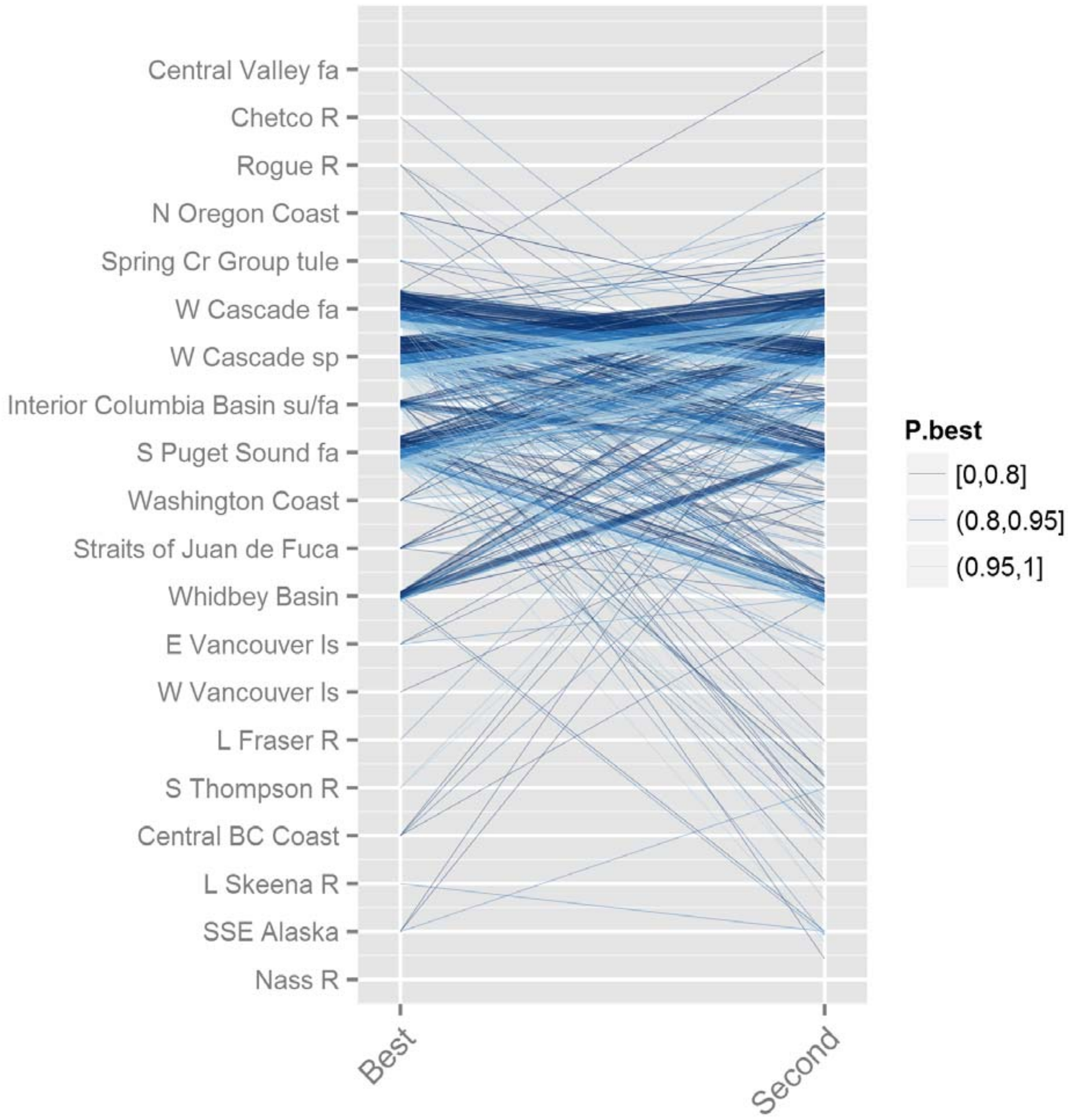

Central Valley fa
Chetco R
Rogue R
N Oregon Coast
Spring Cr Group tule
W Cascade fa
W Cascade sp
Interior Columbia Basin su/fa
S Puget Sound fa
Washington Coast
Straits of Juan de Fuca
Whidbey Basin
E Vancouver Is
W Vancouver Is
L Fraser R
S Thompson R
Central BC Coast
L Skeena R
SSE Alaska
Nass R
Best
Second
P.best
[0,0.8]
(0.8,0.95]
(0.95,1]
